# Wandering about allostery

**DOI:** 10.1186/s13062-024-00502-0

**Published:** 2024-08-08

**Authors:** Maurizio Brunori

**Affiliations:** Dipartimento di Scienze Biochimiche, Presidente emerito della Classe di Scienze FMN, Accademia Nazionale dei Lincei; Professore emerito di Chimica e Biochimica, Sapienza Università di Roma, Rome, Italy

## Abstract

It was a Lucky Strike to be working with Eraldo Antonini on hemoglobin and myoglobin when Jeffries Wyman arrived in Rome in 1961. I found myself connected with a number of creative scientists when the concept of allosteric control was conceived and gifted to the life science community. In retrospect, this was a demonstration of the skill and imagination of a few intelligent scientists that I happened to be close to. Those talents demonstrated the power of creativity as pictured by the motto *“Mens agitat molem”*; a celebration of humanism and intellect that paved the way to novel discoveries in the field of structure function relationships in proteins. I have presented hereby some of the events and the people as emerged from my memory over three decades of exciting scientific life.

## Incipit

*“Not all those who wander are lost“.*

*(JRR Tolkien)*

In April 1961 Jeffries Wyman arrived in Rome for a one week visit that eventually extended to a quarter century, attracted by the scientific talent, the irrepressible enthusiasm and the generous hospitality of Eraldo Antonini, the brilliant pupil of Professor Alessandro Rossi Fanelli, the founder of the Rome Biochemistry School and a worldwide known biochemist for his pioneering work on myoglobin. At the time I happened to be working at the Regina Elena Institute for my experimental thesis in Medicine, but was already accepted as a collaborator of Antonini working on hemoglobin and myoglobin. *De facto* I became from day one Wyman’s assistant and his closest collaborator, a strike of luck since I found myself as an insider while the story of allostery unfolded from birth to maturity and beyond.

The Monod–Wyman–Changeux (MWC) model for allosteric proteins, an elegant and powerful theoretical concept, proved to be a succesful evolution in the field of biological regulation based on a cooperative selective mechanism to allow proteins to cope with the ever-changing physiological demands of the cell. The expression ‘‘*allosteric inhibition*’’ was coined by Jacques Monod and François Jacob in the written comment to the experiments on L-threonine deaminase and their consequences carried out by Jean-Pierre Changeux for his PhD thesis at the *Institut Pasteur.* The MWC model is an important chapter in all Biochemistry textbooks and proved remarkably popular with more than 10,000 citations, close to a record for a theoretical paper in protein science. The rigorous and elegant equations were the result of original thoughts and productive interaction between Changeux, Monod and Wyman (visiting professor at the University of Rome).

This paper is an account of some of the people and the meetings dealing with allostery, partly based on documents but mostly from digging my memory. I hope that the reader will understand my personal bias for hemoglobin, the paradigmatic allosteric protein from the very beginning.

*“And when they ask us what we’re doing, you can say, We’re remembering. That’s where we’ll win out, in the long run”.*

*(Fahrenheit 451, R. Bradbury).*

## Jeffries Wyman: from Harvard to Rome via Paris & Cairo

Born in Cambridge (MA) in 1901, Wyman studied at Harvard and then moved to London for a PhD in Biophysics with A. V. Hill. His lifelong friend John T. Edsall was also working in Cambridge (UK). There Jeffries met Francis Roughton, David Keilin, Joseph Barcroft and Gilbert Adair, who had a profound influence on his subsequent scientific career. Back to Harvard in 1927, Wyman worked initially on the dielectric relaxation of amino acids and peptides in water, and then turned his attention to hemoglobin, which remained his beloved protein for the rest of his life. In 1948 he published in the Advances in Protein Chemistry a fundamental review on heme proteins recapitulating some of the seminal work on homotropic and heterotropic interactions in hemoglobin [1].

**Fig. 1:**
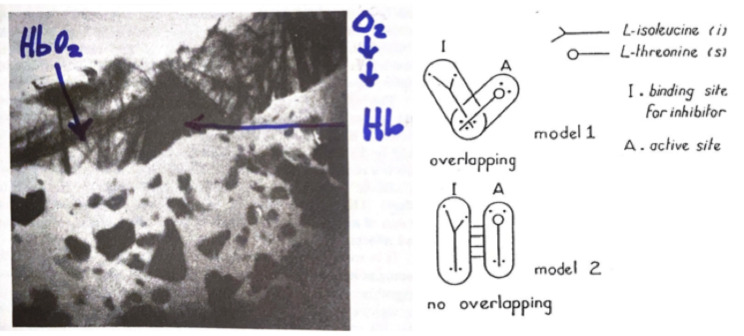
(*left panel*) Felix Haurowitz placed hexagonal crystals of reduced deoxy hemoglobin (Hb) with the characteristic purple color of venous blood on a slide under the microscope. With time, oxygen diffusing under the cover slip dissolved the deoxy crystals which were replaced by monoclinic crystals looking brilliant red as the arterial blood. This implied that the whole hemoglobin molecule changed shape on binding oxygen [3]. (*right panel*) The sketch proposed by Jean-Pierre Changeux to illustrate the concept of non overlapping sites to account for the peculiar binding curves of the enzyme L-threonine deaminase and the control by the feedback inhibitor L-isoleucine [6, 7]

In 1951 Wyman and Allen published in the Journal of Polymer Science a theoretical paper written while Wyman was in Japan as Scientific Attaché to the US Embassy (Jan–Nov, 1950) [2]. They argued for a change of paradigm as clear from this sentence: “.*the reason why certain acid groups are affected by oxygenation is simply the alteration of their position and environment which results from the change of configuration of the hemoglobin molecule as a whole accompanying oxygenation’’*; and ‘‘*if we are prepared to accept hemoglobin as an enzyme*,* its behavior might give us a hint as to the kind of process to be looked for in enzymes more generally*.’’ It is surprising that in connection with such a prophetic statement the authors failed to refer to the remarkable experiment published in 1938 by Felix Haurowitz (a distant relative to MF Perutz) [3]. He demonstrated that deoxy-hemoglobin crystals crack upon oxygenation implying a substantial change in overall shape and surface structure upon binding oxygen. (Figure 1, left). The world had to wait for Max Perutz and coworkers [4, 5] to solve by crystallography the 3D structure of horse met-hemoglobin, and later of reduced deoxy hemoglobin, to show the existance of two different quaternary states of the α_2_β_2_ tetramer.

In 1952, Wyman resigned from Harvard and moved to Paris as Science Attaché to the US Embassy. Over that period he established stimulating connections with several outstanding french scientists working at the *Institut Pasteur* and the *Institut de Biologie Physico-Chimique*; such as Jacques Monod, Andrè Lwoff, François Jacob, René Würmser and others. Wyman had met Monod years before but scientific and personal interactions were established while he was in Paris. In 1956 Wyman moved to Cairo as Director of the Scientific Cooperation Office of UNESCO. Although totally detached from active science, he wrote with John Edsall the first volume of Biophysical Chemistry, a fundamental book for years to come. A profound change in his scientific life arrived in 1961. While in Cambridge (UK) for a short visit, Wyman was introduced by Quentin Gibson to Eraldo Antonini who invited him to Rome for a one week visit. Captivated by Eraldo’s exceptional talent and engaging personality, eventually Wyman decided to extend his visit and indeed lived in Rome for 24 years as a guest professor at the Regina Elena and the Biochemistry Institutes of the University. The best part of his scientific life started anew; at age 60.

At the time I was already working with Antonini on hemoglobin and myoglobin, and became *de facto* Wyman’s assistant, a strike of luck. Quite early we agreed that he would help me with thermodynamics and I would teach him Italian. We began by reading twice a week “*I promessi sposi*”, and progress was quite promising. Unfortunately after a long summer holiday in Boston, Wyman’s italian was back to square one! Since I felt a bit discouraged and he was not eager to be tortured twice a week, we diplomatically dropped the deal and his Italian remained limited, just enough to address a sheppard if encountered in the country during his weakly long walks.

The collaboration between Antonini and Wyman catalyzed a stream of scientists to work in Rome for longer or shorter periods. One of the first was John Taylor from Louisville (Ky), the last PhD of William Mansfied Clark and the first one to publish reliable redox equilibria of hemoglobin [8]. John and his wife Portia arrived early 1963 from England driving an attractive sport/decap MG. His daily presence in the Lab was a *bonanza* as I became his pupil; over and above general advice, I learned to put togheter from scratch a redox apparatus: platinum electrodes, calomel reference cell, anaerobic argon line, titration chamber, chemical mediators, and the proper assembly of a Leeds & Northrup potentiometer and a Rubicon galvanometer. After approx four months of hard work we had completed novel experiments on human hemoglobin which were discussed, submitted and published in the JBC [9]. Happily, these first results paved the way to novel experiments and more papers published before the end of John’s sabbatical.

At one point I was asked for a meeting with Prof Rossi Fanelli, our godfather. Contrary to my anxiety, we had a long conversation on myoglobin and on the life in the Institute, no rules no orders, in a relaxed mood. Towards the end, he told me the story when he had been approached by a famous horse breeder from Argentina with the proposal to inject myoglobin in the muscle of thorough bred horses to overperform. A burst of laughter ended our first proper conversation.

## Jean-Pierre Changeux, une thèse de doctorat avec Monod

During 1958-59 Changeux was striving to identify an interesting topic for his PhD Thesis at the *Institut Pasteur*. After frequent consultations with Monod and Jacob, in the fall of 1959 he decided to work on the functional regulation of the enzyme *L-threonine deaminase* and on the molecular mechanism of activity control by L-isoleucine. He reproduced experiments published by Umbarger, yet on the bench J-P was struggling to overcome the difficulties that biochemists usually faced when attempting to characterize a new enzymatic system, such as erratic activity and aging of the preparation; yet he unveiled reproducible effects of heating or heavy metals on the feedback inhibition of the enzyme by isoleucine. Experiments on the effect of L-valine definitively convinced J-P that L-threonine and L-valine bind simultaneously to the enzyme suggesting distinct binding sites. *Tempus fugit* working hard month after month, while results on other enzymatic systems showing similar functional peculiarities were being published.

At the beginning 1961 Jacques Monod together with François Jacob contributed to the organization of the 26th Cold Spring Harbor Symposium on *Cellular regulatory mechanisms* and invited J-P to give a short presentation of his fresh experimental data on L-theonine deaminase which suggested that functional control was *via* a “non-overlapping” mechanism implying that the active and the inhibitory sites are topographically distinct but linked *via* the protein (Fig. 1, right). The paper presented by Changeux elicited interest such that during the discussion Bernard Davis from Harvard pointed out some analogies with hemoglobin characterized by heme-heme interactions in the binding of oxygen. At the end of the Symposium, Monod presented an overall synthesis of the meeting and in particular the regulatory mechanisms discovered for complex enzymes including of course L-theonine deaminase. During his concluding remarks however, he did not pronounce the term *allosteric control* which appeared only later in the note published with François Jacob in the Proceedings of that Symposium. In a nutshell, the expression *allosteric control* was written for the first time ever in that comment, fixing unequivocally the birth date of Allostery [6].

Monod, Changeux and Jacob [10] developed the concept of allosteric control in a paper written by-and-large in the fall of 1962 and published in the J Mol Biol. Allosteric interactions were clearly defined as indirect control mediated by a conformational change of the protein, possibly involving a change in the state of aggregation of the enzyme in question. In spite of some criticisms, that paper had the great merit of providing generality to the concept that allosteric proteins are essential components of biological control *via* the activity of specific regulatory enzymes.

Wyman immediately appreciated the general significance of the new concept and the powerful message embedded in the definition of *allosteric control* as coined by Monod, Jacob and Changeux. He understood the relevance of allostery to account for homotropic and heterotropic interactions in hemoglobin, as suggested by Bernard Davis (see above). Indeed he wrote a paper entitled *Allosteric effects in hemoglobin* [11] which presented some of the original experiments obtained by the 30 years old Eraldo Antonini: the selective removal by digestion with carboxypeptidases A or B of the C-terminal amino acids residues of the α or the β subunits of human hemoglobin was associated to the loss of heme-heme interactions and of the Bohr effect [12]. This demonstrated the crucial role of the C-terminal residues in the control of hemoglobin’s peculiar functional properties, which was later supported by Perutz [13]. In addition Romano Zito et al. [14] showed that an overall quaternary change controls the differential accessibility of oxy and deoxy hemoglobin to proteolytic attack by the carboxypeptidases. I remember helping Eraldo and Romano in preparing these two enzymes, a complex extraction procedure carried out on a saturday afternoon given that purification demanded treatment of the pancreas with a lot of aceton which saturated the corridor of the Regina Elena Scientific Division in spite of the open windows.

Wyman’s paper was presented at the 1963 Cold Spring Harbor Symp in June; I helped supervising the secretary Cynthia Cook to collect figures, select references and check typos; no intellectual spill over since on that occasion I was only asked to help the helper. At the Cold Spring Harbor meeting in the absence of Monod, Jean-Pierre Changeux presented the work on L-theonine deaminase, later reported in the first draft of his PhD Thesis. In July 1963 a french organized *Colloquium on regulatory mechanisms* took place in Marseille [15]. Monod had decided to reach Marseille by sailing his own boat but arrived too late because of a strong adverse *mistral*. Jean-Pierre was once again the front man of the Pasteur group on allosteric control; the paper, signed by Changeux, Ullmann and Monod, had an attractive title: *“Un modèle plausible de la transition allostérique”* and the layout was by-and-large that of the 1965 paper. Wyman was obviously quoted because of his prophetic work on hemoglobin with D Allen.

## Kendrew and Perutz: the birth of structural biology

It just happened that over the same period, the work carried out by Perutz, Kendrew and coworkers at the Cavendish Laboratory in Cambridge (UK) marked a scientific revolution. *Annus mirabilis* was 1960 for the *Life sciences* when the three dimensional structure of myoglobin and hemoglobin was published in Nature on February 13th [4, 16]. Since the beginning of the century, hemoglobin was considered the ideal versatile model for studying the structure–function relationships in globular proteins. John Kendrew and coworkers solved the 3D structure of myoglobin, the simpler hemeprotein purified from the red muscle of the sperm whale; Max Perutz and coworkers solved that of horse (met)hemoglobin, the more complex α_2_β_2_ tetrameric oxygen carrier characterized by heme-heme interactions and the Bohr effect.

To solve the 3D structure of these two protens was universally considered an impossible task. Indeed for a long time progress in the interpretation of the X-ray diffraction pattern of crystalline globular proteins was marginal at best, and various experimental improvements were inconclusive. Data reported in 1949 by Kendrew and Perutz at the Barcroft Memorial Conference held in Cambridge would have discoraged most scientists. The sustained effort lasted more than 20 years. Around 1953 Perutz and collaborators discovered a trick to solve the *phase problem* by reacting hemoglobin with mercury. This proved to be a terribly difficult endeavour, the saga being described by Perutz in a few passionate pages in his book *“Science is not a quiet life”* [17]. The *isomorphus derivative method* eventually allowed to solve the 3D structure of these two hemeproteins and others to come.

Kendrew and Perutz described the sense of excitement when realized to be the first people on the planet to see the three dimensional structure of a protein. This extraordinary achievement is universally considered the birth of structural biology and secured Kendrew and Perutz the Nobel Prize in Chemistry in 1962. In the next 40 years Perutz and coworkers would continue to work on hemoglobin and improve resolution to unveil the molecular basis of cooperativity, to provide an interpretation of disease in abnormal Hb mutants and to understand the adaptation of oxygen delivery in different species.

**Fig. 2:**
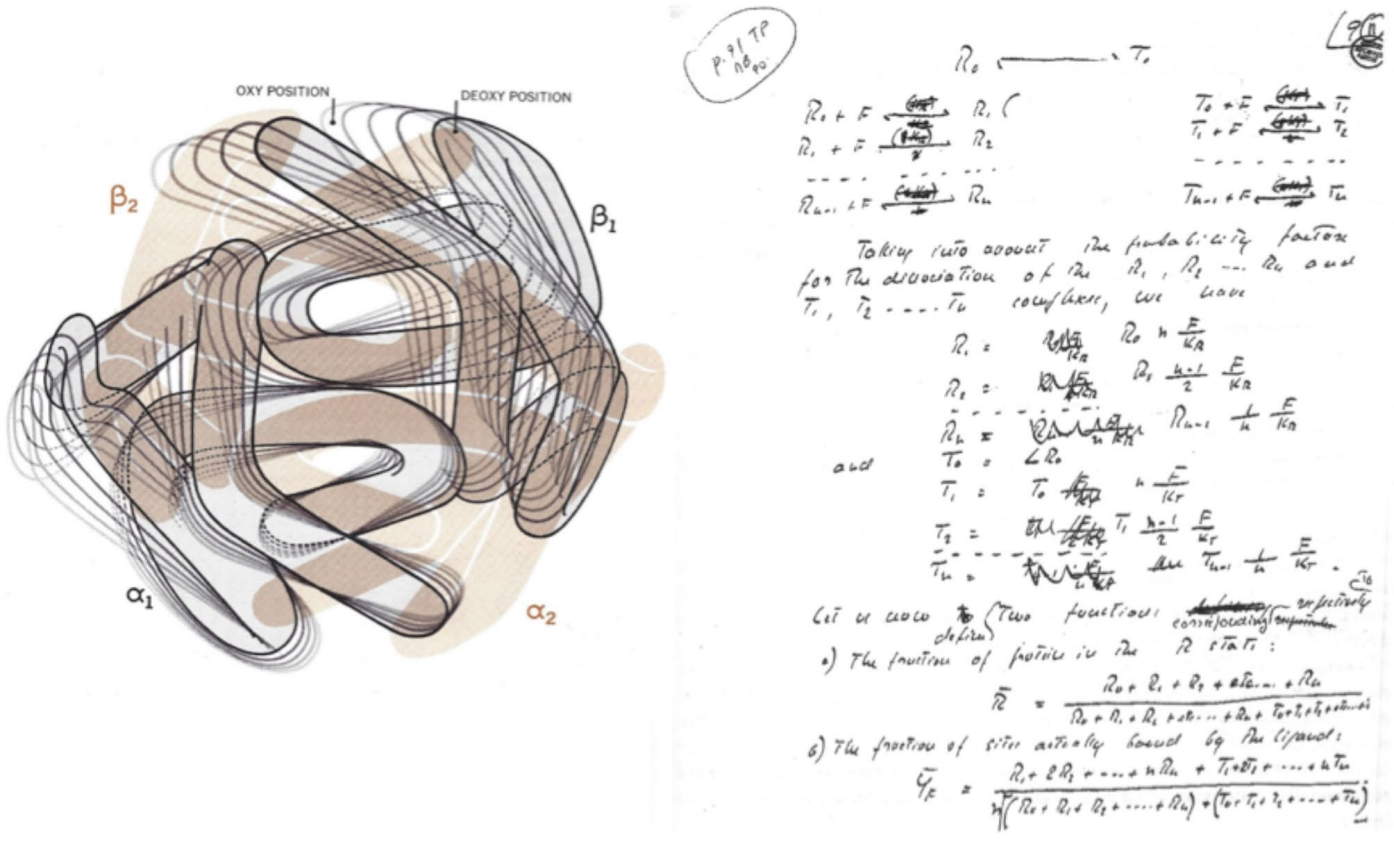
(*left panel*) An artistic sketch of the oxygen linked change in quaternary structure of tetrameric hemoglobin, adapted from [19]. (*right panel*) The overall scheme and the equations of the MWC model, hand written by Jacques Monod himself. (Fonds Jacques Monod, Archives de l’Institut Pasteur). From [15]

In the winter of 1962 Max Perutz was invited at the *Institut Pasteur* for a conference on the 3D structure of hemoglobin. Even with a poor resolution (at 5.5Å), the overall structure of the tetramer showed that the four hemes are separated by 25 to 30 Å and thereby a direct heme-heme interaction mechanism was excluded. The role of the protein proved crucial for a molecular interpretation of homotropic and heterotropic interactions [2], as predicted by Wyman and Allen New crystallographic data on reduced hemoglobin published by Muihead and Perutz [18] proved that deoxygenation is associated to a large change in quaternary structure (Fig. 2, left). Early 1963, Monod was invited to Cambridge for a Conference on regulatory enzymes and allosteric control. I don’t know if he discussed with Perutz about some obvious similarities between the intriguing enzymatic control discovered by Changeux for L-threonine deaminase and hemoglobin’s cooperativity, as suggested by Bernard Davis. In anycase, the different quaternary states of deoxy and oxy(met) hemoglobin were soon taken as the paradigm of the two allosteric states referred to in MWC as the R and the T states.

At the time the functional and structural characterization of hemoglobin was more advanced than that of any other protein. It may suffice to recall some of the scientists that made fundamental discoveries even before the second World War; such as G Adair, L Pauling, JF Roughton, J Wyman and other prophets of protein science. Because of its specific relevance to allostery, I like to recall a pioneering experiment published in 1959 by Quentin Gibson [20] who analyzed the kinetics of binding of CO to deoxy hemoglobin after rapid ligand photodissociation induced by a *Porter/ Norrish flash* apparatus. Partial photolysis of HbCO populated an intermediate called *quickly reacting form* characterized by (i) a 20 fold faster CO rebinding kinetic constant and (ii) a peculiar optical spectrum of the deoxygenated protein. Years later it was demonstrated that Gibson’s quickly reacting form is the allosteric *R-state* as defined by MWC.

## Enhanced collaboration between Monod and Wyman

The sequence of events that progressively led to closer collaboration between Wyman and Monod has been summarized in a very interesting and historically professional paper written by Henri Buc, called *“Interactions between Jacques Monod and Jeffries Wyman*,* or the burdens of co-authorship”* [15]. Buc was himself a young member of the Pasteur group at the time of JPC’s seminal discoveries. To write this account of events Henri consulted many of the documents available at the *Institut Pasteur/Fonds Jacques Monod* including several preliminary drafts of the Monod-Wyman-Changeux paper, the personal correspondence between Monod and Wyman, and JW’s collected letters while in Rome, later donated at the Harvard Library.

On September 25,1963 Wyman paid a visit to Paris for a Seminar on linked functions in hemoglobin. It is interesting to notice that on the 4th of November, Monod handed over to his secretary the first hand-written draft of the paper to be later submitted to the J Mol Biol after changes following conversations and discussions with Wyman, Changeux and other colleagues such as Robert Baldwin who pointed out that the equations defining the state function and the ligand saturation should include the statistical factor because of the four hemes per tetramer. The paper by Henri Buc [15] presents a scheme (see Fig. 2, right) illustrating what was going to be the energy ladder connecting all the ligand saturation levels of the two allosteric states, **R** (relaxed) and **T** (tense).

The Monod-Wyman-Changeux (MWC) model was the outcome of extended discussions that took place during most of 1964. In a paper called *“*Recollections of Jacques Monod” [21] Wyman writes that: *“The paper as it stands was written almost wholly by him (i.e. Monod) and was presented to me more or less as a fait accompli for discussion and criticism”.* In fact Buc [15] writes that Monod and Wyman had an intense exchange of ideas during a good part of 1964, by letter and personal visits. They met in Paris for the celebration of the 50th anniversary of the *Société de Chimie Biologique*, followed by a detailed letter of Monod dated April 24th. On May 1st 1964 Monod came to Rome to discuss face to face with Wyman about the paper and preferred not to deliver a talk at the University. I guess that on the occasion Monod was guest of Wyman in his splendid apartment overlooking Palazzo Farnese, where he lived happily with his wife Olga Lodigensky, her dog Strega and the maid Mafalda.

No one in the Rome group was aware of Monod’s visit, including Antonini who was the driving personality of all original experiments on hemoglobin and the mind behind the extensive results sumarized in the review published in Advances of Protein Chemistry [22]. In March 1964 Eraldo had been invited for the plenary Conference at the first FEBS Congress held in London. He delivered his talk in front of about 1000 biochemists, after presentation by Prakash Datta, the *factotum* of FEBS Letters. The day before we were in Cambridge to discuss with Professor Jack Roughton, Director of the *Institute of Colloid Science*, about the role of CO_2_ in the Bohr effect of human hemoglobin, a topic of interest to Luigi Rossi Bernardi working there. We were supposed to meet at 11,00 *sharp*, but arrived an hour late because of the time change. Prof Roughton was visibly unhappy chewing his tie; nevetheless Eraldo managed very well the discussion on the data. The meeting was productive and we left for London with a complete agreement with prof. Roughton.

After the visit to Rome, Monod wrote to Wyman a warm personal letter dated May 29th 1964 and attached a newly elaborated version of the joint paper that was going to be eventually submitted. Changeux, who had defended successfully his PhD Thesis, contributed to improve the draft with general comments, specific changes and quantitative analysis of the oxygen binding data to sheep hemoglobin received from Lyster and Roughton, and the design of all the figures. Monod was attracted by a possible role of structural symmetry in controlling the stability of the oligomers. Over-and-above the central concept of the theory was the concerted/cooperative nature of the two state model and the role of a conformational selection mechanism that would shift the relative population of the two allosteric states.

***On the nature of Allosteric transitions: a plausible model***, was the final title of the paper submitted on December 24th 1964 to the JMB. In the “Recollections of Jacques Monod” [21] Wyman writes: *“I vividly remember a meeting Jacques and I had with several of our friends at the MRC Laboratory in Cambridge to discuss the manuscript and its suitability for publication in the Journal of Molecular Biology. As I recall it*,* these included Max Perutz*,* John Kendrew*,* Francis Crick and Sidney Brenner*,* a rather formidable group of critics. The attack mainly centered on the symmetry ideas*,* and Jacques bore the brunt of it. But afterwords*,* in the calmer athmosphere at tea*,* we all agreed that the paper*,* symmetry and all*,* should be published as it stood*,* a decision that in retrospect no one could possibly question”.*

When the MWC paper appeared in print [23] I was in Goettingen at the Max Planck Institut für Physikalishe Chemie directed by Manfred Eigen. I was supported by a National Science Foundation grant awarded to Prof Wyman. My local connection was Todd Schuster, a close friend who had been in Rome for stopped flow experiments on the kinetics of release of the ligand linked Bohr protons [24]. In Goettingen we worked hard to characterize the kinetics of oxygen binding to myoglobin and hemoglobin, using the temperature jump machine, only available for us during the night shift [25]. Kasper Kirschner, working on the allosteric enzyme yeast glyceraldehyde-3-phosphate dehydrogenase [26], had a copy of the MWC paper presumably received by Eigen. We had long discussions about the concept of allosteric control, and coming from the Rome hemoglobin group I was an active participant but unfortunately had no privileged information about the close collaboration between Wyman and Monod.

Once back from Goettingen I found that Wyman was in the US. We met later in Naples at the International Biophysics Congress organized by Adriano Buzzati Traverso, an outstanding geneticist, founder and Director of the LIGB (Laboratorio Internazionale di Genetica e Biofisica). During the meeting I had the opportunity to be introduced to Jacques Monod who delivered the general talk on the theory of allosteric control; followed by Wyman who presented more abstract thermodynamics and mentioned some of the recent experiments carried out in Rome. There was a large crowd in the Auditorium including memorable personalities representing the cream of Molecular Biology such as Francis Crick, Manfred Eigen, John Kendrew and many more; and a lot of interesting discussions. Unfortunately Jean-Pierre Changeux and Eraldo Antonini were both absent. In his paper honoring Monod, Wyman [21] recalls that while in Naples, Monod, Kendrew, Crick, Wyman and possibly some other big name had the opportunity for a sailing afternoon in the bay of Naples invited by Crick, who was joint owner of a fine cutter. As an accomplished sailor, Jacques Monod grabbed the tiller and much enjoied the gentle breeze heading towards Capri.

As a matter of fact, the MWC theory attracted much attention and widespread interest including some skepticism. Quite naturally tetrameric hemoglobin was the ideal system to discuss and further investigate allosteric control. Max Perutz had shown that the four hemes are separated by a large distance (25–30 Å) and the protein populates two different quaternary states, the R & T conformers of the two state concerted model (see Fig. 2, right). The MWC paper had a very positive effect in attracting a large number of smart scientists to test the fundamentals of the model. The challenge was amplified by the harsh competition with Koshland’s et al. [27] subsequent alternative model based on the concept of *induced fit*, whereby binding of a substrate to the active site of a subunit induces a conformational change in that particular subunit of the oligomeric enzyme. Thus, contrary to the basic concept of concerted allosteric change, the induced fit is a model with sequential population of all intermediate states. The KNF model that has been especially applied to molecular enzymology, recalled *in nuce* the basic idea proposed by Linus Pauling in the late thirties to account for heme-heme interactions in the oxygen binding to tetrameric hemoglobin [28].

In the following year I attended a set of stimulating lectures delivered by Howard Schachman (Berkeley, CAL), the main speaker at an advanced Biochemistry and Biophysics Course organized in Pisa by Sandro Pontremoli, Rossi and Moruzzi. Schachman was a stellar biochemist, world wide known because of his book on *Ultracentrifuge in Biochemistry*, and later admired for his work with JC Gerhadt on aspartate transcarbamylase, a classical allosteric enzyme [29]. Jocking about the explosive role of the just published MWC model, he presented a figure with the overall time course of recruting young cleaver sudents for a PhD in Biophysical chemistry. Since 1960, the trend indicated a worring decrease in the number of interested voluteers, but Howard predicted that allostery would produce an inversion towards a positive trend in quality.

The year 1965 ended with a fantastic event: **the Nobel Prize for Medicine** was awarded to Jacob, Lwoff and Monod with the following motivation: *“for their discoveries concerning genetic control of enzyme and virus synthesis”*. No formal mention of the allosteric theory, but many felt that it was of help. I could only imagine the grand festival at the *Institut Pasteur* for this extraordinary highly deserved recognition.

## Beyond the Nobel

At the end of 1965 **Jean-Pierre Changeux**, already worldwide famous, went to Berkely for a post-doctoral experience in the Department of Virology where he established with Merry Rubin a productive collaboration which lead to an interesting theoretical paper on allostery dealing by-and-large with the role of heterotropic ligands in controlling hemoglobin’s function [30]. In February 1967 he moved to Columbia University in New York and changed field starting his lifelong passion for receptors. As pointed out by Claude Debrù (personal communication), Changeux had already presented in the conclusion of his PhD Thesis some ideas about the application of Molecular Biology to neuroscience, and speculated about extention of the allosteric concept to membrane phenomena including synaptic transmission. His scientific activity at Columbia was concentrated almost completely to allosteric control of membrane proteins including ligand gated and G-protein coupled receptors. In 1970 he succeded in purifying the nicotinic acetylcholine receptor from the eel electric organ by employing a snake toxin as a trapping ligand. This was the first membrane pharmacological receptor which was purified, and the result paved the way to Changeux’s main interest in modern neuroscience [6, 31, 32].

**Jeffries Wyman** was actively involved in the merry-go-round of experimental work fueled by Antonini’s inventive mind and his extraodinary talent for personally carrying out sophisticated kinetic experiments. Jeffries was arriving daily to his office at the Regina Elena, either with Eraldo’s car or sometimes by motorcycle a quick ride through Rome on the back of my wonderful Ducati 250 cc Monza. Wyman was working on analyzing our experimental data or elaborating on the thermodinamics of linkage and allostery [33]. Over the years, many outstanding people came through the Laboratory at the Regina Elena. to visit Jeffries. When Robert Alberty arrived in Rome for some general discussion on Allostery, Wyman handed over some paper on allosteric control apologizing for the extensive number of equations; and received an immediate reply by Alberty *“I am not scared by complex maths”*. A great *duetto*.

John Kendrew was a frequent visitor starting around 1963 and for several years to come, most probably to consult with Wyman about the inception of EMBO and later when the establishment of the EMBL (European Molecular Biology Laboratory) was being negotiated at the european level. Given the complexity of the endeavour involving governemental decisions, Professor Wyman’s diplomatic talent and natural discretion were precious. Naturally I never asked, and just followed the fantastic achievements of their subtle diplomatic minds. Incredibly just very recently (may 2024) I red an extensive historical account by Francesco Cassata [34] dealing with different events involving the european molecular biology community over the period 1963 to 1970; documents and letters reported in that paper demonstrate the crucial positive role of Wyman while working on hemoglobin. During one of the early visits of Sir John I had the opportunity to present recent data on the reversible thermal denaturation of *Aplysia* myoglobin which lacks the so-called distal histidine; he made some polite comment but enthusiasm was marginal. On another occasion I drove Kendrew and his lady friend Joanne to Sperlonga, a wonderful sea village south of Terracina where Wyman had rented a flat with a beautiful view. Sperlonga at the time was a peaceful place with opportunities to swim and meditate; Wyman was inspired by the view on the Mediterranean as documented in the introduction of the paper on the binding potential [35]: *“In the course of reading at a window by the sea the proofs of a paper*,* I was suddenly struck by the idea…”* a romantic incipit that would have pleased his grand father Professor Jeffries Wyman, the famous Harvard anatomist.

After the Nobel, **Jacques Monod** was even more active in debating about conflicts and interactions between science and society at large. Since the beginning of the decade, he had been one of the members of the group of european scientists involved in preparatory discussions and meetings aiming at creating a molecular biology european network. After intense activities (see also above), this new non-governamental organization the *European Molecular Biology Organization* (EMBO) was launched in July 1964, 60 years ago. Incidentally, Max Perutz was the first President and Jeffries Wyman the first Secretary General. In 1967 Monod was elected Professor of “Molecular Biology” at the *Collège de France* and delivered a plenary conference entitled: *“From molecular biology to the ethics of knowledge”.* As reported by Claude Debrù (personal communication), he discussed the fundamental role of chance in the emergence of sophisticated control molecular mechanisms during biological evolution, and commented on the perspectives for modern medicine. He outlined the philosophical consequences for Society of this novel powerful approach, and commented on the fundamental problem of emerging distrust of science even among educated people, a very serious problem even today.

Monod continued to meditate on profound engaging problems, which he elaborated, extended and presented in his famous book “***Le hasard et la nécessité. Essai sur la philosophie naturelle de la Biologie moderne”***, published in the spring of 1970. Among the many aspects concerning the general philosophical concern of people, Monod clearly emerged as a rational supporter of *reductionism* as the classical intellectual position whereby the study of a complex system must proceed from disaggregation to a subsequent synthesis of the parts. He introduced the term *teleonomia* as an intrinsic property of living creatures that possess a project of functional development, and was convinced that molecular biology would be an important tool to tackle also the complexities of the brain. The book was a great success by any means; I feel that even today Monod’s Essay should be recommended to all students aiming at understanding Biology. After all, the great evolutionist Theodosius Dobzhansky wrote that *“Nothing in Biology makes sense except in the light of Evolution”.*

Throughout his life, Jacques Monod has been deeply concerned with the general problems of modern Society. It is known that during the nazi occupation of France he was an active member of the *Resistance; a*nd across the stormy student rioting period called *May 1968*, he took an intelligent position for the students demands. Given his propensity to be a good citizen, he accepted in April 1971 the responsibility as Director general of the *Institut Pasteur à Paris*.

The irrepressible enthusiasm and clever day-to-day initiative of **Eraldo Antonini** account for the successfull publication record of the Rome group; for example with the characterization of the isolated α and β subunits of human hemoglobin, purified following Bucci and Fronticelli [36]. The non cooperative and high oxygen affinity subunits were shown to quickly recombine to yield a cooperative deoxygenated tetramer, a process associated to optical changes taken as markers of the *quaternary constrain* according to the MWC model. The kinetics of binding and dissociation of different ligands (O_2_, CO, NO, isocyanides etc.), followed by stopped flow, temperature jump and flash photolysys was achieved thanks to the contribution of Milina Chiancone (Eraldo’s *protégé*); and several smart friends such as Gino Amiconi, Johnatan and Beatrice Wittenberg, Annette Alfsen, Morris Reichlin, Quentin Gibson, Bob Noble, Todd Schuster, and others. In due time the isolated α and β subunits would allow one to test directly a crucial prediction of the two state model by preparing artificial intermediates of the type (α^+^CN^**−**^ β)_2_ with two ferric CN^−^ bound subunits per tetramer and two deoxy subunits capable of O_2_ binding. Antonini was a bit skeptical about the MWC concerted two state model based on the argument that if a minute yet finite population of the R-state was present even in the absence of oxygen, it should be possible by addition of a large excess of haptoglobin to trap R_0_ easily detectable by CO binding kinetics. The negative outcome of this experiment was understood much later when Nagel and Gibson [37] demonstrated that haptoglobin binds only to free αβ dimers resulting from dissociation of the oxygenated tetramer, while dissociation of the stable deoxy tetramer is much too slow.

The impact of the Rome group at the time is whitnessed by many international connections and qualified collaborations. Wyman’s charismatic personality and Eraldo’s talents catalysed an intense flux of visitors that came to the Regina Elena and the Biochemistry Institute of the University of Rome. Charles Tanford, Lynn Hoard, Masao Kotani, Geoffrey Gilbert, Annette Alfsen, Quentin Gibson, Bob Shulman, Bob Noble, Guido Guidotti, John Edsall, Morris Reichlin, Kaspar Winterhalter, and others. Rufus Lumry’s sparkling intelligence and original approach to a scientific problem were very stimulating; his sense of humour was engaging and fitted his physical look. Charlie Tanford’s profound knowledge of protein folding and the role of the hydrophobic effect were revealing. Inspired by Rufus, Antonini, Wyman and Professor Rossi Fanelli organized over the years several informal meetings on hemoglobin which became known as the *La Cura Conferences* from the location near Viterbo where the first one was held. The format was totally informal, blackboard and chalk discussions on hemeproteins with the broader picture of protein chemistry in mind; essentially without time limits. Eraldo once recalled Hans Frauenfelder’s talk on myoglobin dynamics and conformational substates that lasted four hours without intervals. When Bob Shulman came to the second *La Cura Conference*, I was in charge of the logistics together with Gino Amiconi; after being shown his bedroom, Shulman looked quite unhappy and on the verge of leaving the meeting. Admittedly the La Cura Castle was an old mansion with comforts somewhat inadequate relative to US standards. In emergency, prof Antonini was called for a negotiation that eventually ended with a good compromise: Bob decided to stay, contributed substantially to the verve of the discussion and came again for other La Cura Conferences in different locations; one of the last being held in Villa Serbelloni at Bellagio, courtesy of the Rockfeller Foundation.

In March/April 1967, approx. one month before the six-days war, I attended a Biophysics school in Israel organized by Ephraim Katchalski and Bernard Pullman. The lectures were initially held in Eilat and later at the Weizmann Institute in Rehovot. This was a fantastic experience as I had the opportunity to listen at extraordinary people such as Shneior Lifson, Gregorio Weber, Ephraim Katchalski and Bo Shulman who delivered a plenary lecture on high resolution proton NMR of enzymes, an absolute novelty for me. Michael Sela was an exceptional host. I was invited in his Office to sign the famous *Sela’s book of visitors*; an emotion to meet one of the authors of the famous 1961 Anfinsen’s paper on protein folding. We were taken to see Marc Chagall’s stained glass windows at Abbell Synagogue, to attend a concert by the Israeli Synphonic Orchestra and to visit the eastern border to overlook behind a fence Jerusalem east, still palestinian territory. Later in the year I went to Urbana (ILL) to work with Gregorio Weber supported by an EMBO fellowship. I appreciated immensely the science and humanity of Gregorio, a pioneer in protein fluorescence; and had a pleasent and productive collaboration with Sonia Anderson. Since Gregorio had just received a powerful nanosecond laser, we carried out photolysis experiments on the Myoglobin-CO adduct but unfortunately missed discovering geminate recombination, helas.

In the second half of 1967 the SIB (Società Italiana Biochimica) received from IUB (Intl Union of Biochemistry) the official offer to organize in Rome the VIII International Biochemistry Congress 1970, a great recognition of the scientific standing of the italian Biochemistry. Professor Rossi Fanelli and his group started with the complex procedure requested for such an important event. Unfortunately in the spring of 1968 the students mouvements flared in California and in Europe especially France. Since the same disorders were expected to begin also in Italy, Rossi Fanelli consulted the Italian authorities who adviced the SIB to withdraw from the event, too dangerous. I remember seating in Rossi Fanelli’s office when the decision was taken; we were all depressed but at least the IUB Council managed to shift the responsability to the Swiss Biochemical Society and the site of the Congress was moved to Interlaken.

In 1968 **Antonini** was appointed full professor of Molecular biology in the Faculty of Science of the University of Camerino. The scientific activity in Camerino had a miraculous reinassance; the project on the structure and function of the hemoglobins from trout began there and quickly flourished thanks to the enthusiasm of young talents, principally Bruno Giardina and Giancarlo Falcioni. In 1970 Antonini was called to the Chair of Medical Chemistry in the Faculty of Medicine, University of Rome; we had just published in the Annual Review of Biochemistry an extensive paper on hemoglobin [38].

## Hemoglobin is propelled centre stage

I don’t know who coined the statement that *hemoglobin could be promoted to the rank of an honorary allosteric enzyme* [39], but I bet it may have been Monod himself. The decades after publication of the allosteric theory were most exciting, productive and very competitive. A large number of outstanding scientists were attracted by the challenge and excited by the availability of more and more detailed information on the 3D structure of different hemoglobins and myoglobins as obtained by Perutz and friends round the world. It is not difficult to understand why hemoglobin became the paradigmatic allosteric protein.

Perutz was captivated by the basic concept of the MWC model and worked very hard in comparing the crystallographic structures of deoxy and liganded hemoglobin. Figure 2, left, is an artisitic sketch of the quaternary changes occurring upon the binding of oxygen to reduced hemoglobin. In 1970 Perutz published in Nature a seminal paper [13] entitled *“Stereochemistry of cooperative effects in haemoglobin”.* He proposed that a few well-identified salt bridges have a fundamental role on the stability of the low-affinity T-state, and envisaged a pathway for the transmission of structural changes from the trigger at the heme iron to the α_1_β_2_ interface, endowed with specific interactions compatible with just two quaternary states. At the active site of deoxy hemoglobin the high spin iron atom is out of the porphyrin plane by approx 0.55Å, and upon distal side ligand binding moves into the porphyrin ring pulling the F helix *via* proximal His.

The fundamentals of this paper were illustrated by Perutz during the opening Conference of the VIII International Congress of Biochemistry in Interlaken (CH). I remember sitting near my dear friend Takashi Yonetani in the grand Auditorium full of biochemists lissening at Max illustrating the 3D structure of oxy and deoxy hemoglobin, the location of the salt bridges constraining the T-state and the active site trigger. A stellar talk. In the discussion I asked if his interpretation was in contrast with the data published by Antonini et al. [12] on human hemoglobin digested with carboxypeptidases A or B; and Max responded by referring to unpublished data by John Kilmartin who was able to dissect the role of the penultimate Tyrosine from that of the C-terminal amino acid in the two subunits (His for the β and Arg for the α).

This seminal work stimulated many new experiments and novel tests igniting what Perutz called *“The haemoglobin battles”* [17]. Since the crystallographic data were collected on horse methemoglobin, it was necessary to prove that the 3D structure of CO bound ferrous hemoglobin was consistent. The out-of-plane position of the ferrous iron by 0.55Å in deoxyHb seemed initially inconsistent with. EXAFS measurements jeopardizing the out/in transition at the heme iron; but after a lot of work Max showed that this opposition was faulty. The complex between globin and cobalt-porphyrin produced a functionally competent Co-globin endowed with cooperative oxygen binding (n_H_= 2.2) and allosteric control in spite of the lower oxygen affinity (by 10 to 25 fold). This novel oxygen carrier prepared initially by Hoffmann and Petering [40] was later investigated by other groups including Petsko, Yonetani et al. [41]. X-ray crystallography of human deoxy Co-globin showed the cobalt to be only 0.33Å out of the plane of the ring, and thereby the ligand linked motion of the proximal His is smaller than in hemoglobin. EPR experiments showed definitely the presence of a H-bond between bound oxygen and the distal His. EXAFS experiments carried out on James Collman’s extraordinary *picket fence porphyrin* compounds [42] supported the crystallographic data. When Boaz Shaanan [43] finally solved the 3D structure of oxy hemoglobin it was confirmed that the iron is indeed in the plane of the ring in both subunits. In the end Perutz was confident that in deoxy hemoglobin the ferrous iron is really 0.55Å out of the plane of the ring and plays the role of trigger.

Kilmartin and Rossi Bernardi [44] had published oxygen binding isotherms of human RBC to clarify the effect on affinity and cooperativity of carbon dioxide, a classical effector in the control of oxygen delivery to the tissues. Reinhold and Ruth Benesh [45, 46] discovered that DPG (2,3-diphosphoglycerate), a component of normal RBC, has a marked effect on the oxygen affinity of human hemoglobin by binding preferentially to the low affinity T-state. The binding site of the 1:1 complex between tetrameric Hb and DPG is in a cavity in the diad axis of tetrameric deoxy hemoglobin. The unequivocal identification of the stereochemically fitting amino acid side chains was demonstrated in 1972 when Arthur Arnone [47] solved the 3D structure of the complex. The effect of DPG has been properly considered fundamental for the physiology of oxygen delivery, and DPG was immediately ranked as the prototype of an allosteric effector.

In a twin paper Perutz [13] presented his hypothesis for the fundamental role of the C-terminal residues in the alkaline Bohr effect. In deoxy hemoglobin a salt bridge between the C-terminal His146(β) and Asp94(β) increases the pK of the imidazole to 8.0; upon ligand binding; the C-terminus is disordered and the imidazole’s pK drops to 7.1. Additional contributions were proposed to come from Val1(α) and His122(α). Kilmartin et al. ([48] found by NMR that the pK of His146(β) in fact decreases upon ligand binding. Perutz admitted a crucial role of the C-terminal residues of the α and β subunits as discovered by Antonini et al. [12] removing the C-terminal amino acids by digestion with carboxypeptidases. It may be of some interest to recall that in 1938 Wyman had proposed a role for two histidines in the Bohr effect based on analysis of the acid–base titration curves of oxy- and deoxy-hemoglobin, and their enthalpy changes [49]; however at the time he incorrectly concluded like L. Pauling that these histidines were coordinating the heme iron.

Several groups raised obiections to the identification of these specific Bohr groups. Among others, a critical position has been defended by Chien Ho and collaborators based on NMR spectroscopy. For a while Chien mantained that the Bohr effect is contributed by many of the 22 His residues each contributing with a small pK shift [50]; a battle that continued for several years with bitter criticisms and conflicting results. I felt that Perutz’s hypothesis was essentially correct and I was definitely convinced once Barra et al. [51] discovered that in Trout Hemoglobin I, characterized by cooperative oxygen binding but no heterotropic effects, all the amino acid residues identified by Perutz and Arnone were either absent or chemically modified. *What else?*

## Hemoglobin complexity is engaging

**Fig. 3:**
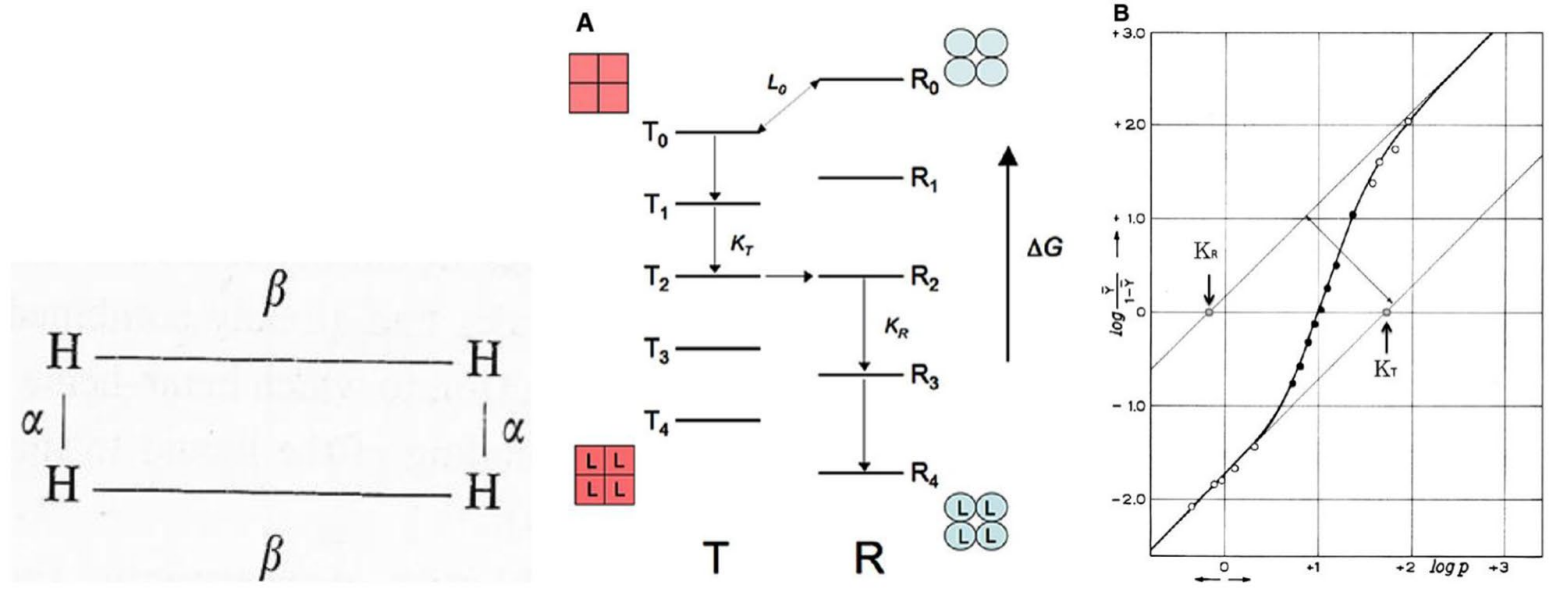
(*left panel*) The rectangular model of Wyman [1]; see also [53]. In the light of now available 3D structures, the vertical sides **α** highlight stronger interactions across α_1_β_2_/α_2_ β_1_ subunits; and the horizontal sides **β** refer to the weaker α_1_β_1_/α_2_β_2_ interactions. Hereby I report a discussion by Wyman [1] when it was only known that hemoglobin is a tetramer. The rectangular model was compared with Pauling’s square model [28]. JW writes: “The four hemes (H) occur in identical pairs, members of the same pair interacting very strongly (dimers connected *via* **α**), members of different pairs (connected by **β**) much less strongly. The interaction between members involves deoxygenated hemes…”. From unpublished experiments in urea (4.5 to 6.0 M; nH from 1.8 to 1.9), analysis returned **β** = 4 and **α** = 400, yielding a stabilizing free energy of interaction ΔF = 3550 cal/heme. Quite remarkable! (*middle panel*) Energetics of the MWC model, and the Hill plot. *Top*: The MWC model postulates that tetrameric hemoglobin populates two allosteric states: T (tense) and R (relaxed), different in quaternary and tertiary structure. The energy level diagram depicts the two quaternary states (squares and circles) and the 10 ligation species (T_0_ to T_4_ and R_0_ to R_4_). The oxygen dissociation equilibrium constants of the two allosteric states are: K_T_ and K_R_; L_0_ defines the population ratio of the two states in the deoxy. tetrameric Hb. For a fully concerted quaternary transition the switch-over point will be at the level T_2_– R_2_ in the case of a symmetric binding curve. (*right panel*) Hill plot of the oxygen equilibrium of sheep Hb in borate buffer and 19 °C; saturation range going from < 1% to ~ 99%. Total free energy of interaction ΔFi = 2.6 kcal/mol heme.; nH = 2.95. (From [53], modified)

As Fogarty Scholars in Residence at the NIH, **John Edsall** and **Jeffries Wyman** organized a meeting on *Allosteric interactions*, which was held at the Stone house on Campus at the end of April 1971. By-and-large the invited participants were all working on hemoglobin starting with Perutz, a unique opportunity to discuss in depth his seminal papers. Being asked to talk, I decided to tackle the *dimer hypothesis* as summarized by Antonini [52] in his paper in Science. Everyone in the audience including myself was convinced that a free αβ dimer per se is incompatible with a Hill coefficient greater than 2.0. I was aware that the sentiment in the audience was against the dimer hypothesis, nevertheless I ventured to present our data on the equilibrium and kinetics of human hemoglobin under dissociating conditions although my after dinner chinese candy recommended: *“do not talk too much”.* It was clear during discussion that I was not very convincing to say the least, partly because I failed to present and discuss *Wyman’s rectangular model* based on the assumption that the α_1_β_2_ subunit interface would store most of the interaction free energy while the complementary α_1_β_1_ interface would be of minor importance, see (Fig. 3, left).

During discussion I was challenged for comments concerning several positions against the dimer hypothesis. Gibson et al. [54, 55], had published experiments on human hemoglobin by stopped flow, flash photolysis and analytical ultracentrifuge to demonstrate that the deoxy dimer is quickly reacting and therefore non cooperative. Somewhat similar data were reported by Kellet and Gutfreund [56] showing that when dilute oxyhemoglobin containing free αβ dimers was rapidly deoxygenated by dithionite, there was no evidence for species reacting slowly with CO. Unpublished experiments from Cambridge [57] demonstrated that carboxypeptidase-B digested human hemoglobin which in the presence of 0.9 M MgCl_2_ is dissociated into dimers is non cooperative in oxygen binding. Last but not least it was recalled that DPG, the paradigmatic allosteric effector, binds only to the tetramer. The consesus was that the low-affinity T-state requires the tetramer; and Quentin closed the issue by stating: “*the dimer hypothesis is dead”.*

In 1969 Shulman et al. [58] had published a paper by the provocative title: *“Absence of heme heme interactions in hemoglobin”* showing that the 4 hemes do not interact direcly. At the Stone House Bob discussed the kinetics of ligand binding to hemoglobin by reference to the canonical energy level diagram as depicted in Fig. 3; and concluded for consistency with the MWC model [59]. The conclusion was that to a good approximation, available ligand binding kinetic information on human hemoglobin is consistent with the predictions of the two state allosteric model. Somewhat later Ogata and McConnel [60] published novel experiments on the binding of spin-label triphosphates to either deoxy or partially CO bound human hemoglobin, as well as to hemoglobin Cheseapeake and half-ligated valency hybrids α_2_^CN^β_2_ and α_2_β_2_^CN^ prepared according to Brunori et al. [61]. Ogawa and Shulman [62] showed that these half ligated hybrids diplay distinct NMR spectra attributed to the α and β subunits in the T and R states; more importantly resonance shifts detected upon addition of DPG indicated that T and R are both populated in the half-ligated state in agreement with predictions of the MWC model [63]. The Stone House meeting at NIH was most challenging but over-and-above I had the opportunity to meet William Eaton and to visit his Laboratory in the basement of Building 2. close to Elliot Charney’s lab. I was curious to understand why Bill wanted to measure optical spectra in hemoglobin crystals using a microscope equipped with polarizers; for the next hour he delivered an extensive clear account. Over the next decades we became very good friends and I discovered that not only he is an outstanding scientist but also passionately fond of tennis.

Two months after the Stone House meeting, the book by Antonini and myself [53] entitled *Hemoglobin and myoglobin in their reactions with ligands* was published. The book contained most of what was known about hemoglobin and myoglobin at the time, and thereby the 5000 printed copies were quickly soldout. Since it proved useful to people working on hemeproteins at large, many Labs had to make copies of the whole book. The first printed copy was officially presented at a small exclusive meeting held in Paris and organized by the *Institut Pierre et Marie Curie*, specifically by Annette Alfsen, Roma Banerjee, Bernard Alpert, Robert Cassoly, and others. It was a very stimulating and profitable opportunity. During discussion Max Perutz, who had a high consideration of Eraldo’s scientific talent, asked why he did not concentrate on analysing in depth the consistency or inconsistency of hemoglobin kinetics with the MWC model expectations; and sollecited Quentin Gibson’s candid opinion on the work by Hopfield et al. [59]. At the end of the day, on leaving the site of the meeting walking down Rue Gay-Lussac towards *Le Jardin du Luxembourg*, Eraldo donated our only copy to an enthusiastic Boris Atanasov who was mourning that he would never be able to buy such a valuable and expensive book. To celebrate the 50° anniversary of the publication of the *Bible of hemoglobin*, Paolo Ascenzi, Andrea Bellelli and Massimo Coletta acted as Guest Editors for a special issue of *Molecular aspects of medicine* [64].

In the fall of 1971 Antonini and Rossi Fanelli organized a special party to celebrate Jeffries Wyman’s 70th birthday. A selected group of friends, including John Kendrew and Max Perutz, were hosted at the XVI century *Palazzo Farnese in Caprarola* designed by the Vignola. The wellcome by Eraldo and several scientific talks were all delivered in the *Stanza del Mappamondo* painted by Giovanni Antonio da Varese; the banquet and a *post prandium* walk in the exclusive park. It was a memorable opportunity to celebrate the 10th anniversary of the arrival of Jeffries and Olga in Rome.

In the same year Joe and Celia Bonaventura started to work at the Biochemistry Institute in Rome. Joe had published with Austin Riggs an important paper on a novel interesting mutant of human hemoglobin called *Hemoglobin Kansas* [65]. The arrival of Joe and Celia (with their kids Marina and Michelle) was socially very positive and visibly improved the mood of the Lab; the two were friendly, generous with everybody and very enthusiastic. After planning togheter a novel experiment, Joe and Celia were immediately on the runaway and practical difficulties were swept away invoking *EGBAR* (**E**verything *is***G**oing *to***B**e **A**ll **R**ight). After Eraldo operating personally the stopped flow had recorded many shots, the oscilloscope pictures were developped, traced, calculated (*mind you*,* by slide rule*) and the data plotted on millimetric paper, everything done by the next morning. At lunch after a joint discussion, we planned a new experiment and off to go. The Bonaventuras were asked to tackle a new project of special interest to Wyman: the determination of CO binding to myoglobin under a continuous light flux as a means to pump energy into the system. Later the same approach was extended to hemoglobin to assess the effect of light energy influx on cooperativity. Of course the project on the hemoglobins from trout started in 1969 at the University of Camerino was intriguing to Joe and Celia, and they suggested to investigate other fish. Around the middle of 1972 Earl and Thressa Stadman (NIH) visited Camerino to deliver five general Biochemistry lectures, a tremendous success. After my talk on trout hemoglobins, I was invited by the Stadmans to write a Review for Curr Opinion in Cell Biology [66]. Our admiration and sincere friendship for Joe and Celia grew over time. Once they left Rome and moved to the Faculty of the Marine Biology Lab in Beaufort (NC), a stream of roman visitors went to work with them. Our close interaction was the seed that eventually matured into the organization of a scientific expedition to the Amazon.

Several smart theoreticians engaged in unveiling the role of the globin in controlling the oxygen affinity of hemoglobin and the validity of the two state model. In 1973 flying back home after a visit to Britton Chance at the Johnson Foundation in Philadelphia, I went to the LMB to talk to Perutz about fish hemoglobins and the interpretation of the Root effect. Unfortunately Max was very busy, but I had the fortune to be introduced to Attila Szabo, a collaborator of Martin Karplus who kindly devoted some of his time to me explaining their fundamental mathematical model [67] to assess the bonding of the ligand to the iron, the role of the tertiary structural changes and their coupling to the quaternary transition. Unfortunately at one point in the afternoon I had to leave Attila to go to Lymington near Plymouth to visit the Elephant Boat Yard at the suggestion of Brit. I intended to negotiate with Mr Richardson the price of a IOR quarter tonner sail boat designed by Britton Chance Jr. Fortunately I managed to become the happy owner of Gretel II; and for two decades I had the pleasure of sailing her in the Mediterranean, thanks Brit! Years later Gelin and Karplus [68] calculated the most likely change in conformation of the heme complex on ligand binding and the path of the motion and dynamics to the subunit boundaries. Ariel Warshel [69] focussed on understanding the origin of the free energy of heme-heme interactions. He confirmed that the high spin iron is too large to fit in the porphyrin hole, and proposed that an important role should be assigned to the steric repulsion between the porphyrin and the proximal histidine imidazole. Ariel calculated that most of the free energy of cooperativity is concentrated in the oxygenated T structure, the strain being mostly in the helix F and in the FG stretch.

In 1973 Max Perutz organized a grand meeting at the Royal Society in London. Very many of the people working on hemoglobin and myoglobin, friends and enemies, attended the meeting. Any attempt to summarize the proceedings would be fruitless. I was amused by the gossip that Reinold Benesh and his wife, the discoveres of the allosteric effect of DPG, were gypses. A significant disagreement was fired by the provocative findings reported by Warner Love on X-ray diffraction experiments of HbS fibers and crystals with data that contradicted previous results. In anycase, the Cambridge group was outstanding and overwhelming; and the whole meeting was very stimulating and fantastic for the selection of the participants and the impeccable organization. Important improvements emerged with the work of Kiyohiro Imai using his new apparatus to accurately measure the oxygen dissociation curve of hemoglobin, keeping under control the formation of ferric hemoglobin. Experiments extended data points at the bottom and the top of the curve allowing reliable estimates of the oxygen affinity of the T and R states. In 1974 Minton and Imai published novel data on the effect of pH and IHP on the affinity of T state human hemoglobin, showing that the variable bottom asymptote of the Hill plot (Fig. 3, right) is compatible only with a three state model [70]. It seemed reasonable that powerful heterotropic ligands (such as IHP) may induce tertiary effects within the T state, recalling the so-called Root effect characteristic of fish hemoglobins. Imai’s machine stimulated many productive collaborations as summarized in his exellent book *Allosteric effects in Haemoglobin* [71].

**Fig. 4:**
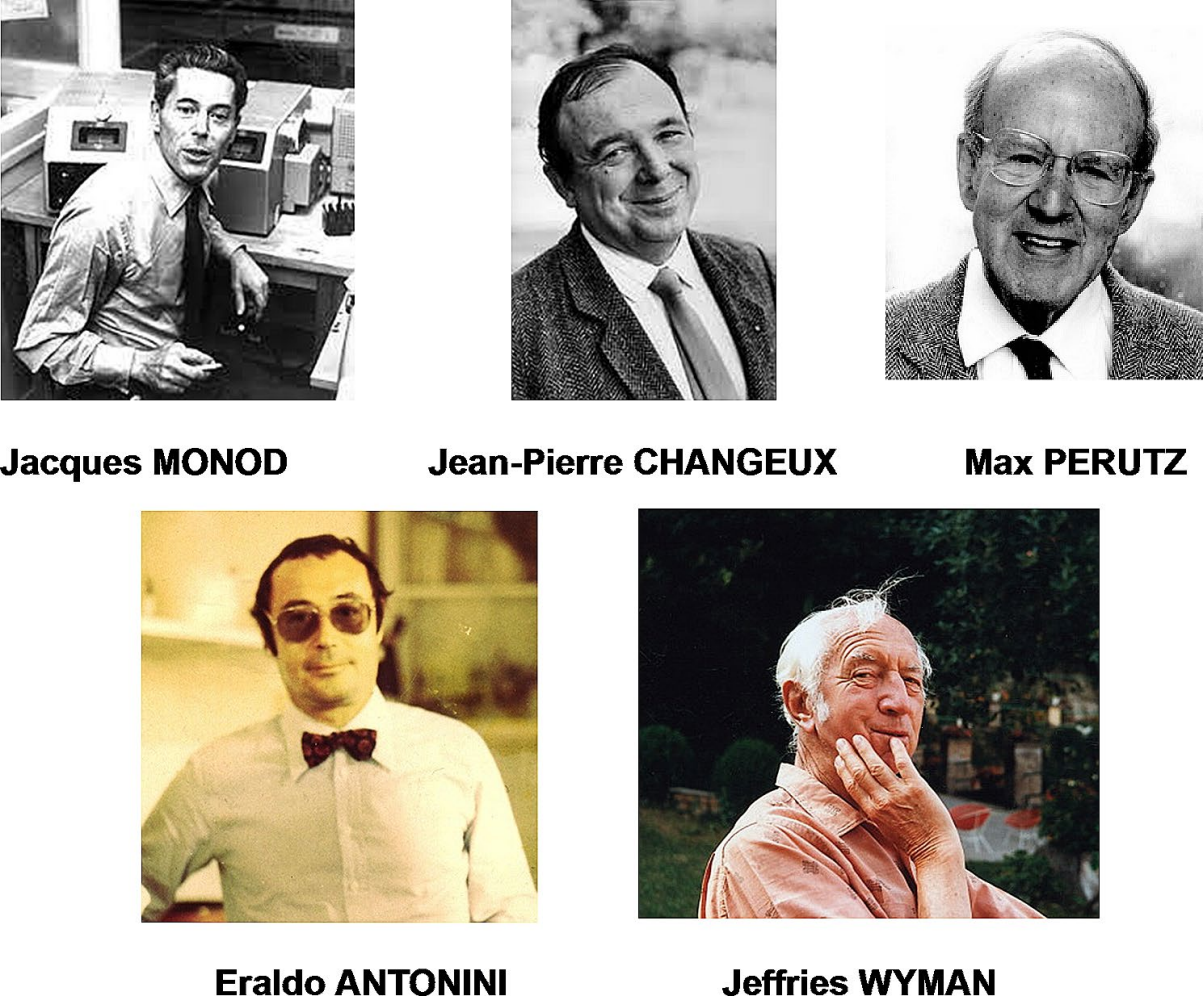
Pictures of Jacques MONOD, Jean-Pierre CHANGEUX, Max PERUTZ, Eraldo ANTONINI and Jeffries WYMAN. (Courtesy Prof M. Brunori)

The kinetics of the quaternary conformational changes in human hemoglobin was characterized by Sawicki and Gibson’s [72] usingy laser photolysis of HbCO to probe of the R—T quaternary change. Analysis of data at different levels of photolysis allowed determination of the rate constant for the R_0_/T_0_ quaternary transition and demonstration that that quaternary rate constant drops by a factor of two for each additional ligand bound. The kinetics was fully consistent with expectations of the MWC model in borate buffer, but at lower pH or in the presence of IHP it seemed that a new rapidly reacting state may be populated.

## From Molecular Biology of receptors to cognition

In 1967 Jean-Pierre Changeux returned to France and in 1972 became attaché to the chair of Molecular Biology held by Jacques Monod. Building on his remarkable experience, Jean-Pierre studied the neurotransmitter signalling mechanisms eventually leading to a breakthrough in the understanding of the human brain. The isolation and characterization of the nicotinic acetylcholine receptor, the elucidation of the primary structure of the subunits and the conformational transitions affected by mutagenesis causing gain-of-function, led to the concept of “*receptor diseases*” [31, 73]. The identification of positive allosteric sites for Ca++, ivermectin and some general anaesthetics inspired a novel drug design strategy. The role of the nicotinic receptor in neuromodulation led to a revolutionary insight into brain signalling and to the design of new categories of drugs [74]. Driven by curiosity Changeux studied the neurotransmitter signalling mechanisms unveiling the multi-scale processing of the human brain, from receptors and synapses to networks and consciousness. Understanding the brain and its pharmacology paved the way to a myriad of clinical discoveries in neurology and psychiatry, from drug addiction to cognitive enhancement, from ageing to Parkinson disease and schizophrenia.

Changeux’s rare talent has, in my opinion, the mark of an original mind. From his seminal work on L-threonine deaminase he imagined that control of enzymatic activity depends on ligand binding at a site distinct from the active site; what he called *non overlapping sites model* (Fig. 1, right). This was the inspiration which paved the way to the MWC allosteric theory. His work enriched our understanding of the brain with the publication of several inspiring books that were widely appreciated, such as *The Nicotinic Acetylcholine Receptors: from Molecular Biology to Cognition* [31, 75]. Changeux has been an independent mind ever since started to work on the PhD Thesis in Monod’s Laboratory. His desire for intellectual independence was recognized and admired by Monod himself, as made evident once, after returning to Paris, J-P found a copy of the famous assay *Le Hazard et la Nécessité* with a dedication by Monod himself reading: *”Au vrai fils spirituel, et comme tel et tout naturellement, un peu parricide”.* Figure 4*depicts the major players of this story.*

## Mutant and abnormal hemoglobins

It was immediately apparent that the possibility to purify and characterize some of the many natural mutants of human hemoglobin was a fantastic opportunity to unveil structure function relationships well before the technology for site directed mutagenesis was available. Only after the mid eightees the first site directed mutants of myoglobin and hemoglobin became available thanks to Springer and Sligar [76] and Olson, Nagai et al. [77]; this breaktrough paved the way to success in tackling detailed molecular mechanisms. However selected human hemoglobin mutants, starting with those defined *abnormal* because associated to disease, proved useful in unveiling the role of specific amino acids in controlling the allosteric properties of hemoglobin.

Hermann Lehmann, a 1938 refugee from Nazi Germany, became eventually full Professor of Clinical Biochemistry in Cambridge, where he started a collaboration with Perutz to correlate the function of abnormal hemoglobins with the perturbation of the 3D structure by the mutant amino acid [78]. The position of each mutated residue was identified with the collaboration of Hideki Morimoto and found to account for the clinical profile based on the perturbation of the MWC allosteric parameters. All the replacements were classified in four categories [78], i.e. the residues in contact with the hemes, those in the contacts between subunits; those in general positions or in external locations (generally asymptomatic). Closer analysis of a selected group of abnormal hemoglobins led to a conclusion that was consistent with the basic framework of the two state allosteric model since an increase in oxygen affinity was associated to the loss of a bond stabilizing the T-state, or vice versa a reduction in oxygen affinity was associated with the loss of bonds stabilizing the R-state. An excellent overview of the abnormal human hemoglobins including all aspects from genetics to structure and pathology, was published in 1983 by Richard Dickerson and Irving Geis [19], in their attractive book simply called *Hemoglobin*. Perutz’s position concerning the consistency of the structural biology of hemoglobin with the two state MWC model has been summarized in the 1989 excellent review paper [79].

**Fig. 5:**
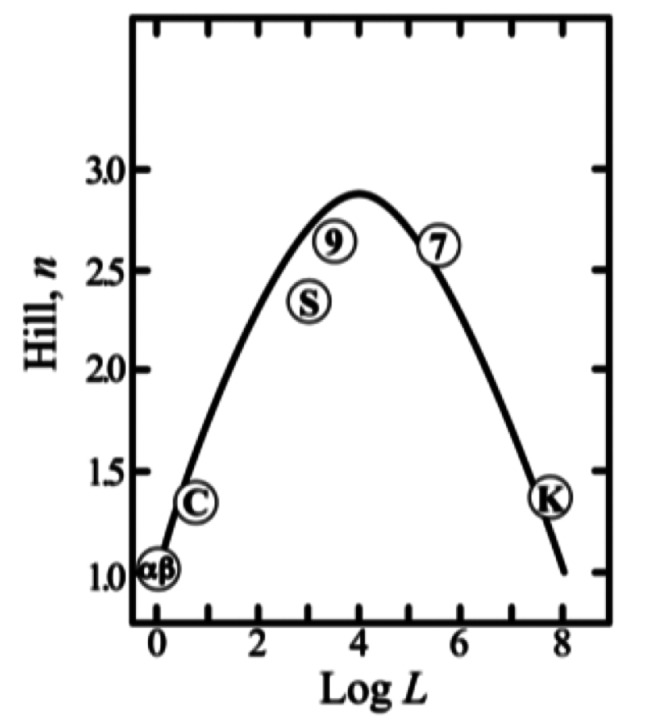
(*left panel*) Correlation between the Hill coefficient n and the allosteric equilibrium constant L. Numbers **7** & **9** indicate data for: human HbA at the two pH values; **α** and **β** indicate the isolated subunits; **S** stands for stripped human HbA; **C** stands for Hb Chesapeake: Arg FG4(92)α1—Leu the mutation interfering with the oxy to deoxy transition; and **K** stands for Hb Kansas: Asn 102(G4)β2—Thr with a destabilized R-state (From [82], with permission)

Bonaventura and Riggs [65] had purified and characterized Hemoglobin Kansas (mutation Asn102(G4)β---Thr), which displays low oxygen affinity, low Bohr effect and reduced Hill coefficient (n_H_ = 1.3). Morimoto et al. [80] later observed that mutation of Asn—Thr at the α_1_β_2_ subunit interface leads to a destabilization of the high affinity R-state. In addition Ogawa et al. [81] demonstrated by high resolution NMR that CO saturated Hb Kansas in the presence of IHP is in the quaternary T-state with normal salt bridges. In 1971 Stuart Edelstein [82] had correlated the allosteric equilibrium constant L_0_ with oxygen binding data for normal HbA, the isolated α and β subunits, and two mutants, i.e. the low affinity Hb Kansas and the high affinity Hb Chesapeake, both displaying reduced heme-heme interactions. The data yielded a bell shaped curve (Fig. 5) consistent with the MWC model; at the two extremes of the bell shape n_H_ approaches unity and oxygen affinity estrapolates to the canonical values of the R and T states.

Sickle-cell hemoglobin (HbS), a single mutant Glu(6)β---Val, is famous because Linus Pauling and Irving Itano [83] proposed that the dramatic deformation of the RBC of the carrier is due to intracellular polymerization of the abnormal HbS leading to hemolysis and thereby anemia. In that paper Pauling introduced for the first time the definition of “*Molecular Disease*”. Many outstanding scientists worked on the mechanism of gelation of deoxygenated HbS and on the structure of the HbS fibers. Polymerization and formation of the fibers occurs only with the deoxy T-state hemoglobin, the interaction between tetramers involving a primary contact between Val(6)β and the hydrophobic EF pocket in the adjacent tetramer. An unexpected observation emerged from time resolved calorimetric experiments carried out at NIH by Ross, Hofrichter and Eaton [84]. The time course of formation of the gel revealed a lag-time of seconds to tens of minutes; the lenght being an inverse function of the HbS concentration to the power of 30, a huge effect.

Based on this peculiarity Eaton and coworkers proposed an original *kinetic theory of gelation* [85]. If the lag-time could be extended, intracellular polymerization of deoxy-HbS can be so slow that the erythrocytes of the carrier can escape the microcirculation and reach the larger vessels before sickling. This would avoid capillary circulation block and thereby painful crisis and tissue damage. Even a small decrease in intracellular HbS concentration would extend the delay time sufficiently to allow more and more erythrocytes to escape the capillaries. Thermodynamic and kinetic experiments in collaboration with Ferrone, Mozzarelli and Coletta [86] supported a double nucleation mechanism and concluded that the velocity of nucleation relative to the RBC capillary transit time is the most crucial parameter [87]. Eaton has been working for 20 years to find a drug that may increase the delay time, to discover an effective and accessible cure. *“The sickled cell. From myths to molecules”* is an interesting book by Edelstein [88] illustrating his personal experience in Africa where in certain areas the incidence of sickle cell anemia was approx. one out of every fifty newborns. Stuart explored possible connections between “endemic” sickle cell disease and the *Igbo* traditional practice to amputate the end of the little finger of a child, a ritual to induce the *ogbanje children* to be reborn. Being involved in the hard work of unveiling the 3D structure of the HbS long fibers, Stuart also describes in the book the molecular abnormalities due to the mutation.

At one point in the seventies Wyman was hit by a severe attack of Menière syndrome immediately diagnosed by our friend Renato Giuffrè. As demanded by this vestibular disfunction, Jeffries were recommended not to move the head to avoid the emergence of new attacks. Therefore he was immobilized in bed for several weeks; no weekend walks in the country. That period concided with an important initiative taken by my dear friends Giorgio Careri (Director of the Institute of Physics at the University) and Paolo Fasella (full professor of Biochemistry in Rome). They were given the task to plan, organize and direct an advanced Protein Biophysics Laboratory in Monterotondo near Rome, completely financed by ENI. This was a unique opportunity to set up expensive very advanced instruments to study the structure and dynamics of proteins, a primary interest of Careri and Fasella. Some of the smart young collaborators of Careri such as Enrico Gratton and Massimo Cerdonio, joined this new Institution. Massimo built a very sensitive megnetometer that was employed to carry out in collaboration with Max Perutz and Bob Noble experiments on human and carp met-hemoglobin which proved crucial to test some aspects of the allosteric mechanism [89].

**Jacques Monod** passed away on 31 may 1976, in Cannes, his last words being: *“Je cherche à comprendre”.* I feel that an engaging fullfilling description of the great man and outstanding scientist is the portrait painted in words by Francis Crick, published in the memorial volume *“Les origines de la Biologie Moléculaire”* [90]: *“Impressionant malgré sa petite taille*,* il imposait l’attention par son intelligence*,* sa clarté*,* sa vivacité*,* et par l’évident ampleur et la gravité de ses intérets. Ne manquant jamais de courage*,* il combinait une simplicité de manières et un humour sarcastique avec un engagement moral profond pour toute cause qu’il jugeait fondamentale”.*

It is tempting to imagine that Monod decided to drift away in Cannes while contemplating the beloved Mediterranean, as depicted by his friend Albert Camus:

*“(…) Oh! Méditerranée! et le miracle de ton histoire*,

tu l’enfermes tout entier dans l’explosion de ton sourire (…)”

Monod’s passion for sailing arose quite late in life but it was intense and challenging. In 2002 I had the opportunity to chat with Agnès Ulmann about the fascination of sailing; and she recalled how the two of them enjoyed the peaceful and emotional night passage close to Stromboli, the boat sloshing under sail and the volcano roaring.

André Lwoff and Agnès Ulmann were the Editors of the volume *“Les origines de la Biologie Moléculaire”* [90] containing 32 papers by stellar scientists and close friends of Monod. The book published in 1980, aimed at providing a portrait of the scientist and of the man, and his fundamental role in the birth of molecular biology. *“Naviguer avec Jacques”* is the title of the contribution by Francis Crick highlighting the passion of a genious for sailing. I am priviledged to have a copy of this volume with a dedication by Agnès Ullmann *elle mème*, given to me on the occasion of the one day meeting held in january 2002 at the Accademia Nazionale dei Lincei, to remember Jacques Monod.

## Biological electron transfer

In the seventies the scientific interest of **Eraldo Antonini** and his coworkers had shifted to biological electron trasfer and energy transduction, a timely problem of fundamental interest for Biophysics and Bioenergetics. He attracted to Rome for extended collaborations Bo G Malmstrom from Göteborg and Colin Greenwood from Norwich. A prompt success was a stopped flow kinetic investigation of the reaction of cytochrome c with cytochrome c oxidase; the results published in Nature [91] clarified that electron transfer from the reduced substrate to the four metal centers of the enzyme is a cooperative process. That started an exciting period of original experiments on the oxidase that continued and expanded thanks to the arrival in Rome of Mike Wilson, his wife Angela and their four months old twins Ruth and Emma. The presence of Mike in the Lab was a blessing which has continued for some decades. A natural talent, a knowledgeable biochemist and a friendly human being full of humor stimulated collaborations and attracted younger people. In 1977 we carried out a kinetic experiment with a double mixing protocol to probe a predicted intermediate of cytochrome c oxidase. I remember clearly that burst of joy shared with Eraldo, Colin and Mike, while looking toghether at the transient on the screen of the oscilloscope proving that the experiment was a success. This result unveiled a new reactive state of the enzyme which we called *pulsed oxidase*; our paper was accepted by Britton Chance for the PNAS [92]. Somewhat later, we were informed that Helmut Beinert from Madison, a *guru* of cytochrome c oxidase, reported on the discovery of *pulsed oxidase* during his opening Conference at the international Congress on redox enzymes in Australia. Naturally we were quite happy of that recognition given that *pulsed oxidase* helped rationalizing several scattered puzzling observations. In addition our finding came just at the time of the fundamental discovery by Martin Wikström [93] that cytochrome-c-oxidase is a *bona fide* proton pump, and thereby makes a direct contribution to the overall energy conservation in the mitochondrial respiratory chain.

Wikström’s discovery fired a fairly harsh intellectually exciting competition with Peter Mitchell. The father of *Chemiosmotic theory* recognized with the Nobel in Chemistry, did not agree with Wikström’s interpretation [94]. These brilliant scientists defended their ideas for years; we followed an entertainig duel at the 1978 *La Cura Conference* held in Caprarola (near Viterbo). A little later at the IUPAB Congress in Kyoto the organizers scheduled two successive talks by Wikström and Mitchell on the question: *Is* c*ytochrome-c-oxidase a proton pump?* We were generally convinced that Marten was the winner, a great discovery that added another important dimension to the physiological role of this complex metallo enzyme crucial to mitochondrial energy conservation.

**Fig. 6:**
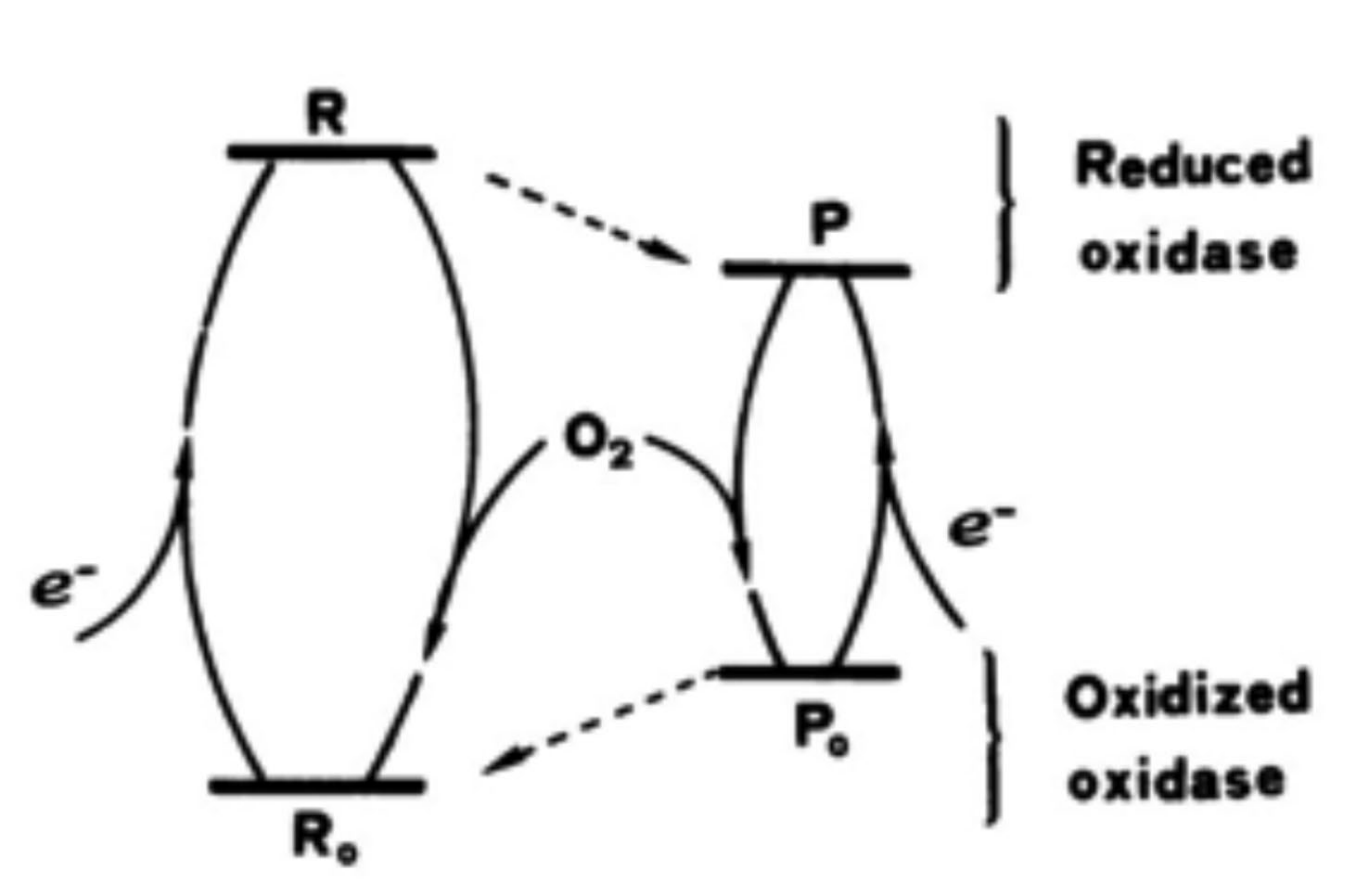
Diagrammatic representation of the two-state model applied to cytochrome-c-oxidase. The relative stability of the two states (Resting and Pulsed) in the fully reduced and in the fully oxidized states is indicated by the position of the energy levels connected by the broken arrows. Oxygen binding to the reduced enzyme and electron donation from cytochrome c are indicated. (From [96])

When cytochrome-c-oxidase reconstituted in small phospholipid vescicles was demonstrated to be fully functional, we engaged in new kinetic experiments investigating the mechanism of coupling between internal electron transfer, the rate control by the transmembrane electrochemical gradient and proton pumping. After some computer simulations and deep discussions involving Antonini, Colosimo and Wyman, a theoretical paper based on the application to oxidase of the MWC model was finalized (Fig. 6). The paper entitled: *A Plausible Two-State Model for Cytochrome c Oxidase.* [95, 96] seemed consistent with the kinetics and particularly fit in showing the general validity of the allosteric concept. At the time we were not aware that this was going to be the last paper actively contributed by Eraldo himself.

## The scientific expedition to the Amazon

The National Science Foundation USA agreed to support a scientific expedition to the Amazon basin on board of the RV *Alpha-Helix*. The project was to study fish bloods and hemoglobins of some of the approx 3000 different species of fish living in the Amazon; and to exploit the rare opportunity to compare hemoglobins from air-breathing and water-breathing fish. The idea of organizing such a fantastic endeavour emerged around 1975 during a dinner in Rome with Austin Riggs, Bob Noble and Joe Bonaventura. Before the *gelato* Joe proposed *out of the blue* to collaborate on writing the necessary application; and having registered our enthusiasm, highlighted the application summarizing: the scientific rationale, the people involved, the budget and the best period (*November and December 1976*, the low water season). In a nutshell, Joe had done the hard part of the work with fantastic *nonchalance;* but declared that my duty was to convince Wyman to join the expedition. This was the easiest of all jobs since Wyman accepted enthusiastically and came with us to the grand mother river.

The two months on the *Alpha-Helix* was unique, exciting and scientifically productive. The ship was equipped for Biochemistry and Physiology experiments with standard laboratories, a cold room, refrigerated centrifuges, spectrophotometers, chromatographic equipment and even a Gibson–Durrum-stopped flow for the study of rapid reactions. Among the many species studied, we were most interested in comparing blood and hemoglobins from fish belonging to the same superorder such as *Osteoglossum bicirrosum* a water breather with usual swim bladder, and *Arapaima gigas* an air breather having modified a super vascularized swim bladder to act as a rudimental lung. Other fish such as *Pteroglyptis pardalis* would use as a rudimental lung their stomach by swallowing air. The 33 papers with the results obtained on board were published in Comparative Biochemistry and Physiology, 1979 vol. 62. Austin Riggs [97] was an admirable Chief Scientist; some of the people on board such as Morris Reichlin, Charles Phelps, Aldo Focesi, Bob Noble, Mike Wilson and Joe Bonaventura had been working in Rome. An important aspect of the expedition was the extended deep discussions held in the ward-room of the ship anchored in the Rio Solimoes about 20 miles from Manaos. The introductory paper written by Wyman [98] entitled *“Variations on the theme”*, was very fit, the theme being hemoglobin’s allosteric control and the variations dictated by the specific physiological requirements imposed by the survival in a wild environnement.

Before Christmas 1976 all our friends left Brasil to go home except for Jeffries, Jeane Davis and myself. We decided to take a tour in the forest and after consulting with the US Consular authority in Manaos, Jeffries contacted Mr Kurt Glück, a german guide likely escaped from Europe after the world war. We took off with Kurt’s motorized canoo wandering for 5 days in the tributaries of the Rio Negro, resting in the natives shelters, meeting the families and sleeping in hammocks; a dream trip documented by my photos. Back in Manaos and Belem, I flew to Rio and than back to Rome on the night of the 31st of december 1976. On the huge jet 4 passengers and 10 hostess. In Rome I found that Federica had taken good care of our family; luckely without need for my advice since communication from the Amazonas was *quasi* impossible.

In 1977 Wyman was elected foreign member of the Accademia Nazionale dei Lincei, proposed by professor Alessandro Rossi Fanelli who at the time was Secretary of the Class of Science. He was very pleased for this recognition and delivered a general conference in the Auditorium of the Villa Farnesina: no slides, just blackboard and chalk. He reviewed allosteric linkage and extended to the thermodynamics under steady state perturbation intrigued by the Bonaventura’s experiments on CO binding by myoglobin and hemoglobin under continuos illumination as a means to pump energy into the system [99]. The talk elicited the curiosity of several fellows and in particular Gaetano Fichera, a senior outstanding mathematician. Wyman’s talk paved the way to a productive collaboration with Fichera and Maria Sneider leading to a complex theoretical paper published in the Proceedings of the Lincei Academy, in 1977.

In the following year Wyman left Rome for a long trip to Papua New Guinea, all by himself. During the preparation of the trip I realized why sometimes before he had asked Carleton Gajdusek (Nobelist for the discovery of the infectious agent of Kuru) to come for a visit. I remember meeting Gajdusek in Wyman’s office at the Regina Elena Institute while he was summarizing his Science papers (still in my office) with the discovery of transmission *via* a slow virus, some photos of the aborigenes and details of his expedition. Fortunately back in Rome after a couple of months Wyman described some of the experiences of living in the wild with the aborigenes. I remember in particular how he was amused in recalling that while sleeping in the hammock, the papua kids would come close to touch his white hairs and run away laughing at that absolute peculiarity. I know that Jeffries wrote a diary about the trip and gave it to Helen Pringle to type, but I never received a copy.

In April **1980** a grand meeting on *Hemoglobin and oxygen binding* with the participation of ~ 250 scientists ranging from leaders to PhD students was held at Airle House in Virginia. Max Perutz participated actively to the sessions dealing with heme proteins and allostery. The 27 papers submitted by the participants were published in 1982 by Elsevier North Holland (New York) in a very valuable volume edited by Chien Ho, Eaton, Collman, Leigh, Margoliash, Moffat and Scheidt [100]. Looking at this book today one is impressed by the outstanding quality and the interdisciplinary blend of the scientific contributions. To facilitate discussion and provide a background for the audience each session was introduced by a plenary lecture as follows: *Heme oxygen bonding* by Martin Karplus; *Origins of cooperative oxygen binding by hemoglobin* by Max Perutz; *Dynamics of oxygen binding* by Quentin Gibson. The latter session was a parade of picosec to millisec myoglobin and hemoglobin dynamics investigated by experiments and computer simulations. Based on photolisys of CO hemoglobin by a (30nsec) laser pulse Duddel, Morris and Richards [101] discovered *geminate ligand recombination.* They observed that a fraction of the photodissociated ligand rebinds to the heme iron within 300 nsec being temporarely trapped in the protein matrix. This paved the way to a huge number of exciting discoveries on protein dynamics investigated by the most advanced laser spectroscopists such as Hochstrasser, Friedman, Spiro, Renzepis, Alpert and others. This area of Biophysics had been opened by Hans Frauenfelder and coworkers [102] introducing the concepts of conformational substates and energy landscape from an analysis of myoglobin ligand rebinding kinetics at low temperatures. We had to wait many years to have crystallographic evidence that indeed the dissociated CO is trapped in the distal cavity of myoglobin; as demonstrated in 1994 by Ilme Schlichting and coll [103] by ultra-low temperature X-ray diffraction coupled to laser photolysis. The role of protein defects in controlloling the ligand trajectory was predicted by Case and Karplus [104] using molecular dynamics simulation. The four Xenon binding cavities were identified by Petsko and coworkers [105] using NMR. Years later with the advent of site directed mutagenesis, the role of matrix cavities in controlling ligand binding dynamics and allosteric behaviour was extensively explored [77, 106, 107].

In **1981** the Congress of IUPAB (*International Union of Pure and Applied Biophysics*) was held in Mexico City. As a member of Council I proposed to organize a special Symposium on the *Biophysics of allosteric control* to honor Wyman’s 80th birthday. The proposal was approved and a number of outstanding speakers were invited. In a packed Auditorium with Wyman in the first row, Kurt Wüthrich, Secretary General of IUPAB, introduced the rationale of the initiative in the light of the MWC model, and I referred to an ad hoc recent statement by President Ronald Regan: “*I believe in Linkage*”. The *parterre* was first class, the talks excellent and the discussion very incisive. In closing I quoted a sentence written by Wyman in one of his classic papers: *Hemoglobin is like a beautiful women: fascinating and ever so attactive* [108].

Spring 1982 **Eraldo** felt very hill and I quickly realized that he had a nasty lung cancer. Renato Cavaliere, his doctor and a very close friend, decided in accord with the family to hide the truth and continued to let him believe that the diagnosis was for a rare malignant form of tuberculosis. I was in Somaliland to teach the summer Biochemistry course at the Medical Faculty in Mogadishu, a compulsory duty being within a formal agreement between Italy and Somaliland. While there, I had no direct information since phone connections were not available, but received two letters with very negative outlook from Milina Chiancone who was desperate. Once back in Rome the situation was not improved and Eraldo was constantly under pain killing drugs. I went quite regularly to his bedside in Via di Parione and we spoke at length about the Institute, the science and the future of younger people including Giovanni. When he passed away on 19 March 1983 I was visiting Mike Wilson in Brightlingsea where I received the bad news from my wife Federica. A very large crowd attented Eraldo’s funeral at the Chiesa Nuova in Rome. The premature death of Eraldo was a blow that struck like an earthquake family, friends and biochemists worldwide. Prof. Noris Siliprandi from Padova, President of the SIB (Soc Italiana Biochimica), proposed that the annual Congress of the Society will forever begin with an *“Antonini Lecture”.* This has been the case as recently summarized by myself [109]. With Milina and Jeffries I wrote an obituary for TiBS [110], and the last sentence was written by Jeffries himself: *“Those who have enioyed meeting Eraldo at work and at home*,* the two focal centers of his life*,* will never forget his irrepressible enthusiasm*,* joy of life*,* friendliness*,* and hospitality. Looking back on him now that he has gone*,* they will perhaps recognize the temper of a Renaissance Italian in his ever questing spirit”.*

## Epilogue

*A*fter Eraldo’s death, the mood in Rome changed. Wyman had to quit his office at the Regina Elena and was offered an office in the Biochemistry Department of the University. In fact for a while we shared Eraldo’s office and I enjoyed people coming to discuss their data with the Professor, when available. Sometimes he would work in his office at the Accademia Nazionale dei Lincei, a very pleasent location close to Piazza Farnese where he lived just across the Tevere. Nevertheless, for all of us the atmosphere was not the same, and eventually in 1985 Wyman left Rome for good and moved to Paris to live with his wife Olga and her sister Marina in a flat in *Avenue La Motte Picquet.* In the summer, they would go to a country cottage in Haute Borne. Several friends such as John Edsall, Joe Bonaventura, Alfredo Colosimo, Enrico Di Cera and others including myself visited Jeffries in Haute Borne. He was particularly happy of the collaboration with Stanley Gill, professor at the University of Colorado who started to work with the Rome group years before when he finished building an original and very accurate method to measure the oxygen dissociation isotherm of hemoglobin. In the mid eighties Stan convinced Jeffries to collaborate in writing a book on *Binding and linkage*: the writing took a while to complete but was eventually published on the 15th of january 1990.

In the same year Olga passed away and I decided to go to Paris to present my condoleances to Jeffries and Marina; the ceremony was according russian tradition and she was buried in the russian cemetery of Sainte-Geneviève-des-Bois. On that occasion I met Wyman’s daughter Anne; this was our first encounter since during the 24 years that Jeffries lived in Rome, Anne came only twice and for short. Jeffries died at home in Paris on 4 november 1995. Among the many recollections of the man and the scientist, I appreciated especially the spirit of the thoughts written by Jean-Pierre Changeux: *“A very moving book for somebody who*,* like me*,* had the priviledge of collaborating with Jeffries Wyman; a fair and deep testimony about the intellect of an immensely important scientist and respected humanist.”* At the funeral, I had the opportunity to chat with his son Jeffries jr and some of the friends and collaborators that worked with the professor.

In June 1999 Anne Wyman came to Rome for two weeks or may be more. She was in the process of writing a *memoir* of her father, therefore wanted to meet and talk with some of the people that had been close collaborators of Wyman for a quarter century, the period which, according to Sir John Kendrew was the most exciting and prolific of his whole academic life. Anne was a learned interesting and pleasent lady that Federica and myself enjoyed very much while she was guest at our home. In spite of her vivid intelligence she may have missed capturing in her book the deep significance of the roman life for her father. Jeffries was enthusiastic about the people and the science in the Lab, and fell in love with the Country and the eternal city, so beautiful and rich of history and art. Very often after work he would walk back home by himself, selecting different paths through the center in search of new beauty.

Anne’s *memoir* was published in 2012 by Protean Press in Rockport, Mass [108]. For me the book was somewhat revealing as I discovered facets of Professor Wyman’s personality that I had missed in spite of having been very close to him for so long. The incipit of the book explains the title **Kipling’s cat**: **“***only the cat refused to become her servant. … when the moon comes*,* he is the cat that walks by himself*,* and all places are alike to him. Then he goes out to the Wet Wild Woods or up to the Wet Wild Trees or on the Wet Wild Roofs*,* waiving his wild tail and walking by his wild lone.” My Father was just like that.*

## Coda

**Fig. 7:**
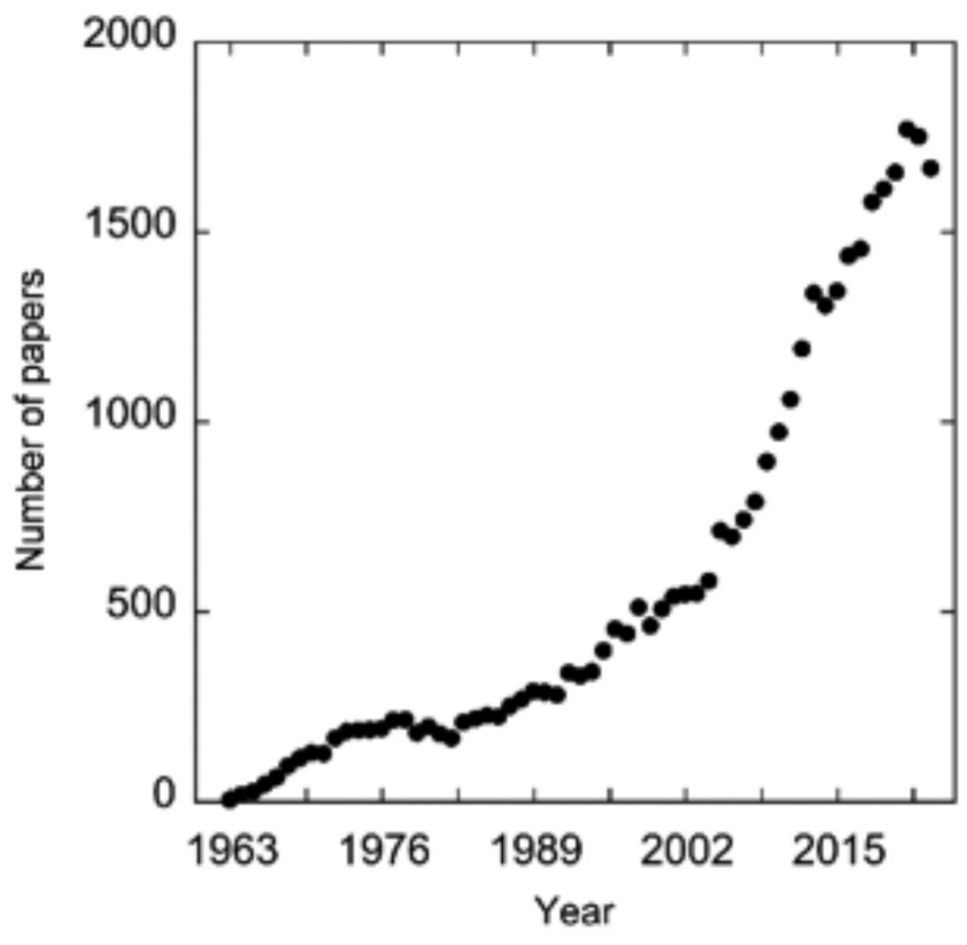
A plot of the number of published papers containing the words “allostery” or “allosteric” in their Title or Abstract. (source Scopus). (Courtesy Prof S. Gianni)

People wondering why the MWC allosteric model has enjoyed such an enormous success for so many years may want to consider the following points: (i) the concept of selection among functionally distinct conformational states of the protein being consistent with the principle of biological evolution; (ii) the formal elegance of the model based on simple mathematics enhancing predictive power; (iii) the introduction of the greek-derived term *allosteric*, a compact description of a complex molecular phenomenon, and a product of classical education. Over the last five decades the number of publications on the structure and function of allosteric proteins from different living creatures has continued to increase. Figure 7 depicts the time course of the number of published papers containig the words “*allostery*” or “*allosteric*” in their Title or Abstract (source Scopus). Notice the sharp increase around 2004, the year of FDA approval of the first *allosteric drug Cinaclet*, indicated for the treatment of secondary hyperparathyroidism in people on dialysis for chronic kidney disease and hypercalcemia in people with parathyroid carcinoma. (Courtesy of Prof Stefano Gianni, Sapienza University of Rome).

The MWC model has been extended over the last few decades to monomeric functional proteins populating (at least) two conformational states even in the absence of ligands and involved in transport, recognition or control. The principle of a ligand linked population shift has been exploited to describe sophisticated structural and functional data obtained on monomeric proteins with widespread functional repertoire [111]. In the case of allosteric monomers the use of the term “cooperative” should probably be avoided being coined to indicate homotropic interactions among several binding sites. Moreover it seems improper to mingle the reader by emphasizing that the ligand-linked population shift of conformational variants implies a *“new view of allostery”*. Indeed this was the founding concept of the MWC model complying with the basic rule of natural selection and with general thermodynamic principles. In their excellent Review on “*Allostery and cooperativity revisited*”, Cui and Karplus [112] commented that “*the most important effect of emphasizing the originality of the new view was to revive an interest in allostery which*,* for several years*,* had been a neglected field of Biophysics”.*

Remarkable progress in sophisticated genetic, biochemical and physical technologies applicable to the life sciences paved the way to exciting findings extending the domain of interest and the structural resolution of proteins to unexpected limits. The progress in novel information on the structure and function of hemeproteins alone has been so overwhelming that I declined several offers to write a modern version of the Antonini & Brunori 1971 [53]. In closing I briefly recall discoveries in two areas of allosteric phenomena that I have followed with curiosity.

In the study of human hemoglobin the approach pursued by William Eaton and his Italian colleagues to characterize hemoglobin trapped in a crystal or in silica gel proved attractive [113, 114]. Accurate measurements of the oxygen dissociation curve in T-state deoxy crystals of hemoglobin showed no apparent cooperativity (n_H_=1), very low affinity, and no evidence for the breaking of salt bridges, as expected from previous data in solution on HbKansas and on fish hemoglobin(s). A small inequivalence in affinity between the α and β subunits in the T-state is compensated by some cooperativity within the αβ dimer recalling some feature of the so-called cooperon model [115]. These important observations have been extended by laser time resolved spectroscopy leading to an attractive mechanism including tertiary contributions [116], consistent with the classical two state quaternary linked allosteric model.

Jean-Pierre Changeux’s multidisciplinary approach led to the discovery that the *nicotinic receptor* (nAChR) is an allosteric membrane protein of great complexity, and clarified the *molecular mechanism of action of nicotine both as a drug of abuse and a cognitive enhancer*. This promiscuous functionality seems to be of great significance for the brain organization at different levels of complexity, from molecules to imagination.

The biochemical and biophysical characterization of the nicotinic receptor has helped our understanding of the multiple roles of this membrane protein whose complexity is demonstrated by being both a ion channel and the target of nicotine [31, 75]. The work has revolutionized common knowledge on the mechanism of action of drugs on the brain. Biophysical experiments revealed the allosteric transition of a typical ligand gated ion channel as the fundamental molecular mechanism of action and control of nicotine [117–119]. This led to the introduction of the concept of *allosteric modulation* and thereby catalyzed a paradigmatic shift in neuropharmacology with the discovery of a novel category of agents binding to allosteric modulatory sites. The introduction of the concept of allosteric pharmacology had a major impact in the field of drug discovery and revealed new ways to unveil higher brain functions. In 2015 Changeux, Brunori and Eaton organized a meeting on *“Allosteric pharmacology”* held at the Accademia Nazionale dei Lincei in Rome.

Changeux’s early findings [7] paved the way for new approaches to investigate brain plasticity connected to the action of nicotine, with insights at the molecular, cellular and network levels. The new outlook is based on the evidence that the binding of nicotine to the nAChR can act both as cognitive enhancer and addictive drug, implying that the allosteric properties of this important receptor may reveal control functions of the brain in health and disease. Interestingly it was discovered that nAChR is involved in aging and in neurodegeneration suggesting a possible role of nicotine and nicotinic derivatives in combatting neurodegenerative diseases.

## Data Availability

No datasets were generated or analysed during the current study.
